# Incidence of Crown and Root Rot in *Rhododendron simsii* Caused by *Phytopythium vexans* in China and Screening of Endophytic Bacteria for Biocontrol

**DOI:** 10.3390/microorganisms13112417

**Published:** 2025-10-22

**Authors:** Zhuo Liu, Yang Sun, Zhuoma Yongcuo, Xiaorui Zhang, Guibin Wang, Yuhua Liu, Tingting Dai

**Affiliations:** 1 Co-Innovation Center for the Sustainable Forestry in Southern China, College of Foresty and Grassland, College of Soil and Water Conservation, Nanjing Forestry University, Nanjing 210037, China; 2240100016@njfu.edu.cn (Z.L.);; 2 Jiangsu Vocational College Agriculture and Forestry, Jurong 212400, China

**Keywords:** *R. simsii*, plant disease, oomycete, *P. vexans*, phylogenetic analysis

## Abstract

*Azaleas (Ericaceae)* are among the most diverse ornamental plants, celebrated for their cultural and economic significance. *R. simsii* has been extensively utilized in horticulture as a parent species for both “pot azalea” cultivars and various cultivars grown in the warmer regions of China. From 2021 to 2023, approximately 15% of *R. simsii* in nurseries situated in the Xuanwu District, Nanjing, exhibited symptoms of wilting and chlorosis. Investigations revealed that these symptoms were caused by a pathogen responsible for crown and root rot. Strains were isolated from the roots of affected plants. The morphology of the colonies was predominantly radial to stellate, characterized by intercalary and terminal hyphal swelling. The sporangia appeared spherical, pyriform, or ovoid with a single papillae. For accurate identification, the 28S rDNA gene (Large subunit, *LSU*), cytochrome oxidase subunit I *(COXI*), and cytochrome oxidase subunit II (*COXII*) genes were amplified through PCR and then sequenced. The species was identified as *P. vexans* after completing the phylogenetic analysis. Healthy *R. simsii* plants were infected with zoospores and developed symptoms similar to those of natural infection. Furthermore, the morphological characteristics of the isolates from the experimentally infected plants were similar to those of the original inoculated strains. This study identified *P. vexans* as the pathogen causing root rot in *R. simsii.* During the sampling process, several strains were isolated from the rhizosphere soil of healthy rhododendron plants. Based on this, research was immediately initiated to explore whether there are specific bacterial species in the soil that have the potential to inhibit the occurrence of root rot. Additionally, an endophytic bacterial strain BL1 was isolated from rhizosphere soil and subjected to Whole-Genome Shotgun (WGS) sequencing, thus constructing a bacterial genome framework for this isolate. The strain BL1 was identified as *Bacillus licheniformis*. To our knowledge, this is the first report of the occurrence of *P. vexans* causing crown and root rot of *R. simsii* in China. In this study, we also focused on exploring the potential of biological control agents against *P. vexans*. The isolation and identification of the endophytic bacterial strain BL1 (*Bacillus licheniformis*) from the rhizosphere soil of healthy soil show strong in vitro antagonism, identifying it as a promising candidate for future biological control studies of root rot in *R. simsii*. The genomic component analysis and coding gene annotation of BL1 provide insights into its genetic makeup and potential mechanisms of action against pathogens. However, these findings are based on in vitro assays. Therefore, further research, including in planta experiments, is essential to confirm the efficacy of BL1 in controlling *P. vexans* infections in *R. simsii* and to evaluate its potential for practical application.

## 1. Introduction

*R. simsii*, commonly known as the Japanese azalea, is a member of the Ericaceae family of shrubs. This species is indigenous to Japan, China, Korea, and various regions of Eastern Asia. *R. simsii* has gained popularity as an ornamental plant widely cultivated in gardens due to its strikingly beautiful flowers [1,2,3]. Flavonoids are bioactive components with analgesic, antimicrobial, and anti-inflammatory properties, with the ability to spit out phlegm, relieve coughs, and protect the cardiovascular system [4]. Beyond its medicinal and ornamental allure, *R. simsii* offers additional utility. The leaves and flowers of various rhododendrons can be distilled for their aromatic oils [5], the bark and leaves can be processed to extract tannins [6]. The symptoms of rhododendron root rot are characterized by the roots or the base of the stem turning brown, with some diseased plants showing water-soaked areas [1]. At the same time, the leaves gradually lose their green color. As the disease progresses, the rot spreads throughout the entire root system, causing the leaves to wilt and curl. Eventually, the entire plant will die. Among them, the root rot caused by oomycetes is the most widespread.

In 2007, Alvarez et al. reported that *R. simsii* seedlings in northern Spain were annually afflicted by leaf spot disease caused by *Phytophthora hibernalis* [7]. Rytkonen et al. isolated several Phytophthora species, including *Phytophthora ramorum*, *Phytophthora cactorum*, *Phytophthora plurivora*, and *Phytophthora pini*, on rhododendron leaf blight in a large nursery in Finland from 2004 to 2007, in 2009, and in 2010 [8,9,10]. In 2011, Tsopelas, P. et al. observed the occurrence of rhododendron leaf blight in a nursery in Greece and identified the pathogen as Phytophthora nicotianae [11].

Many cultivated and natural plants worldwide can be infected by *Phytopythium* species, which causes severe losses in agriculture and destroys natural forest ecosystems. Increasingly reported, pathogens like *Phytopythium helicoides* and *P. vexans* cause severe agricultural losses (e.g., red raspberry [12], kiwifruit [13], corn [14], strawberry [15] and damage natural forests [16,17,18,19]. Global control costs can reach $10 billion annually [20]. *Phytopythium* species are distinguished by variable sporocarp shape (oval to spherical), often with a conspicuous papilla that develops during maturation. *Phytopythium* causes significant diseases, such as crown, stem, root, and collar rot, affect a variety of herbaceous and woody plants [21,22,23,24,25,26,27]. Eradicating these pathogens demands both labor and economic resources. According to the data, treatment costs can reach up to 10 billion US dollars annually [20].

With the increasing awareness of the economic and ecological impacts of these pathogens, ongoing research and development of control strategies are essential to safeguard plant health and productivity worldwide [28]. Plant pathogens cause various crop diseases, resulting in economic losses. The use of bacterial preparations is an excellent choice to combat plant pathogens and is also an excellent alternative to the use of chemicals that are aggressive to the environment and human health. Since *P. vexans* causes multiple plant diseases and there are currently no effective control measures against this pathogen, *P. vexans* was selected for the screening of biocontrol bacteria.

During the investigation of rhododendron diseases in the campus of Nanjing Forestry University, Xuanwu Lake Scenic Area and Zijin Mountain Scenic Area from 2019 to 2022, the root rot of Rhododendron mucronulatum was discovered. This disease led to the death of the entire plant. The symptoms were characterized by water-soaked brown spots on the roots, surface rot and peeling of the root and stem, wilting and drying of branches and leaves, and eventually the death of the whole plant. This disease has had a significant impact on the ornamental and economic value of rhododendrons. The main objectives of this study are as follows: (1) to isolate and identify the causal pathogen using morphological and multi-gene phylogenetic analysis; (2) to fulfill Koch’s postulates and confirm the pathogenicity of the identified isolate on healthy *R. simsii* plants; (3) to screen and isolate endophytic bacteria from the rhizosphere of healthy plants for antagonistic activity against the pathogen; (4) to genomically characterize a promising antagonistic bacterial strain and analyze its potential mechanisms of action.

## 2. Materials and Methods

### 2.1. Disease Investigation, Sampling, and Isolation

Disease-causing crown and root rot affecting approximately 15% of *R. simsii* was discovered in a nursery located in Xuanwu Lake, Nanjing. The plants exhibited symptoms of wilting and chlorosis. Twenty affected plants were collected from the nursery. Microscopic examination was performed using an Olympus BX53 microscope (Olympus Corporation, Tokyo, Japan) revealed that 65% of the root segments contained mycelium morphologically similar to that associated with Phytophthora-producing bacteria.

Initially, the collected samples were washed with water, and the diseased root tissue was then cut into small pieces (approximately 2 × 2 mm) using a sterilized scalpel. The tissue sections were sterilized by immersion in 75% ethanol (Sinopharm Chemical Reagent, ShangHai, China) for 60 s, followed by a 90-s immersion in a 1% sodium hypochlorite (NaClO) solution (Sigma-Aldrich, St. Louis, MO, USA). The soaked tissue sections were rinsed three times with sterile distilled water (filtered through a 0.22 µm Millipore filter (Merck KGaA, Darmstadt, Germany) and blotted dry on sterilized filter paper (Whatman, Leeds, UK)., with each rinse lasting between 1 and 2 min. The water was then dried using sterilized filter paper. The dried tissue sections were subsequently plated on 10% V8-PARP juice agar. V8-PARP juice agar consists of 20% V8 juice (containing 20% V8 juice and 0.2% CaCO_3_), 1.5% agar, 20 mg/L pimaricin (Shanghai YuanYe Biotechnology, Shanghai, China), 125 mg/L ampicillin (Thermo Fisher Scientific, Waltham, MA, USA), 10 mg/L rifampicin (Beijing Solarbio Science & Technology, Beijing, China), and 20 mg/L pentachloronitrobenzene (PCNB) (Dr. Ehrenstorfer, Augsburg, Germany) [29,30]. All isolation steps were performed in a Class II Biological Safety Cabinet (Thermo Fisher Scientific, Waltham, MA, USA). The prepared samples were then incubated in the dark at 25 °C in a model MIR-154-PA incubator (Panasonic Healthcare, Tokyo, Japan) for 3 days. Colonies of isolates could be observed growing on the V8-PARP juice agar medium. A single mycelium was carefully selected from the periphery of the colony using a sterile inoculation needle and then transferred to fresh V8 juice (containing 20% V8 juice and 0.2% CaCO_3_) medium and purified. The purified strains were cultured in darkness at 25 °C and obtained after 3–5 days. All isolates were preserved in the Plant Pathology Laboratory of Nanjing Forestry University.

### 2.2. Pathogenicity Tests

The spore suspension of the isolated and purified strains was prepared for the pathogenicity test on living *R. simsii*. The mycelial blocks of the isolated strains were placed in 10% V8 liquid medium for liquid culture. After three days, mycelial tufts grew out. The mycelial tufts were retained, and the V8 liquid medium was replaced with sterilized tap water, with 3–5 drops of soil extract added (the preparation of soil leaching solution involves mixing 5 g air-dried and sieved healthy soil sample with sterile water at a solid-to-liquid ratio of 1:10. The mixture is then placed in a constant temperature shaker and shaken at 25 °C for 1 h. After centrifugation, the supernatant is collected and filtered through 0.45 μm and 0.22 μm filter membranes to obtain a clear filtrate.). The water was changed every 24 h, and 3–5 drops of soil extract were added each time, until a large number of sporangia were produced and released zoospores. The zoospore suspension was then prepared, with the concentration adjusted to 106 per mL. Two-year-old healthy *R. simsii* were taken out of the pots, and the roots were rinsed with tap water to remove the soil. Wounds were created on the roots using a sterile inoculation needle. The wounded roots were then immersed in the prepared 50 mL zoospore suspension with a concentration of 106 per mL for 2 h to allow the spores to fully invade the roots. Finally, the zoospore suspension was mixed into the potting soil. Three plants were inoculated for each strain, with sterile water as the control group. The plants were then replanted into their original pots and kept moist. The experiment was arranged in a Completely Randomized Design (CRD) within a greenhouse set at 26 °C, with a 12-h light/12-h dark cycle, and watered as needed. The disease severity of the rhododendrons was observed and recorded daily. The entire experiment was repeated three times independently.

After the diseased samples are thoroughly rinsed under running water and air-dried naturally, tissue blocks measuring 2–3 mm in size are excised using sterilized scissors. These blocks are subsequently surface-disinfected by immersion in 75% ethanol for 30 s, followed by immersion in 1% sodium hypochlorite solution for 90 s. The tissue blocks are then rinsed three times with sterile distilled water, each rinse lasting 1–2 min, and excess water is removed using sterile filter paper. The disinfected tissue blocks are then transferred onto V8 medium supplemented with PARP and incubated in the dark at 25 °C for a period of 3–5 days. After the colonies of fungi have grown, purify them according to the method described in Section 2.1, a comparison with the original pathogens was then performed following Koch’s postulates [29].

### 2.3. Morphological Identification

Sterilized hole puncher was employed to obtain mycelial disks from the margins of three-day-old colonies, which were subsequently inoculated onto solid V8 medium [31,32]. The cultures were incubated in darkness at 25 °C, and the morphological characteristics of the colonies—including mycelial color, texture, and growth patterns—were systematically observed and documented. For isolates exhibiting oomycete-like morphology, 20–30 fragments of 2 mm × 2 mm mycelial blocks were excised from three-day-old cultures on solid V8 medium using a sterile scalpel and transferred into V8 liquid medium. Following three days of incubation under dark conditions at 25 °C, mycelial masses were harvested. The V8 liquid medium was then replaced with sterile tap water, and 3–5 drops of soil extract were added. The water was refreshed every 24 h, with the addition of 3–5 drops of soil extract each time, until abundant sporangia formation and zoospore release were observed. The morphology of the conidiophores and conidia of the three isolates were observed under an Axio Imager A2m microscope (Zeiss, Oberkochen, Germany). Microscopic slides were prepared and examined under a Zeiss fluorescence microscope to assess mycelial morphology, sporangia, zoospores, oospores, and other relevant structures [33,34].

### 2.4. DNA Extraction, Amplification, Sequencing, and Phylogenetic Analyses

Each isolate was cultured on V8 solid medium at 25 °C in the dark for three days. Subsequently, 6-mm-diameter mycelial disks were obtained from the margins of the purified colonies using a sterile puncher and transferred into 10% V8 liquid medium for further incubation. The growth of several mycelial clusters was observed within 3–5 days. The mycelium was then filtered and collected. The mycelium water was carefully pressed dry with sterilized filter paper, and then the mycelium was placed in a frozen mortar pre-cooled in liquid nitrogen and pulverized into a fine powder using liquid nitrogen. The fungal genomic DNA was extracted from the aerial mycelia of 5-day-old cultures using the cetyltrimethylammonium bromide protocol [35].

The PCR amplification products were submitted to Shanghai Jielie Biotechnology Company for sequencing. The resulting sequences were analyzed via BLAST 2.16.0 against the NCBI database to identify strains with the highest sequence homology. Relevant literature was reviewed to select appropriate reference sequences, including related target sequences and outgroup genes, which were subsequently downloaded. These downloaded sequences were then combined with the experimentally obtained sequences. Sequence trimming and alignment were performed using BioEdit 7.7.2 software. Trimmed multi-gene sequences were concatenated using the Concatenate Sequence function in PhyloSuite. Following concatenation, the optimal evolutionary model parameters were determined using ModelFinder 2.3.5 within the same software [36]. A maximum likelihood (ML) phylogenetic tree was constructed using IQtree 2.3.5. The resulting tree was visualized and structurally adjusted using FigTree 1.4.4 software, exported as a PDF file, and further edited and formatted using WPS Office.

Initially, the ITS1 and ITS4 primers were used to amplify the internal transcribed spacer (ITS) regions within ribosomal DNA (rDNA), and the sequences were obtained [37]. The primers NL1 and NL4 were used to amplify the large subunit (LSU) rDNA gene fragment [38]. For the cytochrome c oxidase subunit I (COXI) gene fragment, the primers OomCoxI-Levup and FM85mod were used [39,40]. Primers FM58 and FM66 were used to amplify the cytochrome c oxidase subunit II (COX-II) gene fragment [41]. After sequencing, the preliminary identification of pathogens was performed through BLAST 2.16.0 searches in the NCBI database [42] in Table 1 and Table 2. The sequences of the primers and PCR conditions are given in Table 3. The primers were obtained from Nanjing Jinsilui Biotechnology Co., Ltd, Nanjing, China.

### 2.5. Isolation and Screening of Antagonistic Bacteria

Within such habitats, bacteria are more likely to develop efficient antagonistic mechanisms as a result of adapting to adverse environmental conditions and competing for limited ecological niches. Take 10 g of soil (Longitude: 79.913534 Latitude: 37.112149) from the sieved fine-grained soil sample and add it to 90 mL of sterile water. Place it in a constant temperature shaker at 30 °C and 200 rpm for shaking and culture for 0.5 h, which is the soil suspension diluted 10^−1^. Take the soil solution that has been diluted to 10^−1^. Use a sterile pipette to draw 1 mL of the soil suspension and add it to a test tube containing 10^−2^ sterile water. Shake and mix well to obtain a 10^−2^ concentration soil dilution solution. Repeat the above steps to prepare the 10^−3^ to 10^−7^ series of soil diluents in sequence. In the dilution plating method, 100 μL of soil suspensions at concentrations of 10^−3^, 10^−4^, 10^−5^, 10^−6^, and 10^−7^ were, respectively, selected and uniformly spread onto the surface of LB solid medium (1% Tryptone (10 g/L), 0.5% Yeast Extract (5 g/L), 1% NaCl (10 g/L), pH 7.0–7.2). Each concentration was set up in triplicate. The inoculated plates were incubated in an inverted position at a constant temperature of 28 °C. Upon the appearance of single colonies, representative colonies exhibiting distinct morphological features were selected and subjected to streaking on fresh LB plates for the purpose of purification and further cultivation. The entire experimental procedure was conducted in triplicate.

Oomycete inhibitory activity was evaluated using the plate confrontation assay. A three-day-old culture of *P. vexans* was obtained, and a mycelial plug (6 mm in diameter) was excised using a sterile puncher [30]. This plug was then placed at the center of a V8 agar plate. The bacterial strains to be tested were inoculated onto the medium surface using a cross-streaking method, at a distance of 2 cm from the edge of the fungal disk. The plates were subsequently incubated in darkness at 28 °C for 5 days. Antagonistic effects of the tested bacterial strains against *P. vexans* were assessed by visual observation. We tested all the strains for antagonistic properties. We screened out an isolate with a significant inhibitory effect. The experiment was repeated three times.

The endophytic bacteria were inoculated into LB liquid medium and incubated at 160 rpm and 30 °C for 12 h. The resulting bacterial suspension was subcultured under identical conditions for an additional 12 h. Subsequently, 1 mL of the suspension was transferred into a centrifuge tube and centrifuged at 12,000 rpm and 4 °C for 5 min. The supernatant was carefully removed, and the bacterial pellet was resuspended in 100 μL of double-distilled water (ddH_2_O). The suspension was then subjected to a heat treatment at 100 °C for 5 min, followed by freezing at −20 °C for 10 min. This heating and freezing cycle was repeated once. Finally, the sample was centrifuged again at 12,000 rpm and 4 °C for 5 min to remove cellular debris, and the resulting supernatant was used as the extracted genomic DNA of the endophytic bacteria.

The extracted 16S rDNA was amplified via PCR using the universal primers 27F and 1492R [43] targeting bacterial 16S rDNA. Each PCR reaction was performed in a 50 μL volume, consisting of 19 μL of double-distilled water, 2 μL of genomic DNA (100 ng/μL), 2 μL of each primer (10 μmol/L), and 25 μL of Taq DNA polymerase mixture (5 U/μL) (Takara Bio, Kyoto, Japan). Detailed information regarding the primers and PCR amplification conditions employed in this study is presented in Table 4. The resulting PCR products were submitted to Shanghai Panshino Co., Ltd., Shanghai, China for DNA sequencing using the same primers (27F and 1492R [43]). Each region was sequenced five times to ensure data accuracy.

### 2.6. Genome Sequencing

The Whole-Genome Shotgun (WGS) sequencing strategy was adopted to construct libraries of different inserted fragments. Using Next-Generation Sequencing (NGS) technology and based on the Illumina NovaSeq sequencing platform, Paired-end (PE) sequencing was performed on the library. The original raw data were filtered using fastp 0.23.4 software to generate high-quality data. The second-generation data were reassembled from scratch using SPAdes 3.16.0 software, and scaffolds with a length greater than 500 bp and an average depth greater than 10 were screened as the assembly results. Build scaffolds and contig. Single-base correction was carried out using Pilon 1.24 software to obtain the assembly results. By comparing the genomic Sequence with the Nucleotide Sequence Database (NT), the species information of the genome is obtained. The basic characteristics of the genome (such as genome size) were estimated by using the read information of short insertion fragment libraries (library insertion fragments less than 1500 bp) obtained through sequencing. After analyzing the assembled scaffolds using checkM 1.2.2 software, the protein-coding genes of the bacterial genome were predicted using GeneMarkS v4.2 software. Finally, the orthofinder 2.5.4 software was used to conduct genetic family analysis on the sample protein sequences. The software version information is as follows: fastp v0.23.1: https://github.com/OpenGene/fastp (accessed on 23 March 2024), spades v3.15.4: https://github.com/ablab/spades?tab=License-1-ov-file (accessed on 23 March 2024) Hifiasm v0.18.5-r500: https://github.com/chhylp123/hifiasm (accessed on 23 March 2024) Flye v2.9.1-b1781: https://github.com/fenderglass/Flye (accessed on 23 March 2024) Unicycler v0.5.0: https://github.com/rrwick/Unicycler (accessed on 23 March 2024) Necat v0.0.1: https://github.com/xiaochuanle/NECAT (accessed on 23 March 2024) circlator v1.5.5: https://github.com/sanger-pathogens/circlator (accessed on 23 March 2024) diamond v2.0.14.152: http://github.com/bbuchfink/diamond (accessed on 23 March 2024) hmmer v3.4 http://hmmer.org/ blast v2.13.0: https://blast.ncbi.nlm.nih.gov/Blast.cgi (accessed on 23 March 2024) GC_Depth v0.7.17: https://bio-bwa.sourceforge.net/bwa.shtml#1samtools (accessed on 23 March 2024) GC_Depth v1.16.1: https://github.com/samtools/samtools?tab=readme-ov-file (accessed on 23 March 2024) jellyfish K-mer v2.3.0: https://github.com/gmarcais/Jellyfish/releases (accessed on 23 March 2024) genomescopev2.0: https://github.com/schatzlab/genomescope (accessed on 23 March 2024) checkM v1: https://github.com/Ecogenomics/CheckM (accessed on 23 March 2024) GTDB v2.3.2: https://gtdb.ecogenomic.org/ (accessed on 23 March 2024) Pilon v1.24: https://github.com/broadinstitute/pilon?tab=readme-ov-file (accessed on 23 March 2024) GeneMarkS v4.32: http://topaz.gatech.edu/GeneMark/ (accessed on 23 March 2024).

## 3. Results

### 3.1. Symptoms Observed in the Wild State

**Figure 1:**
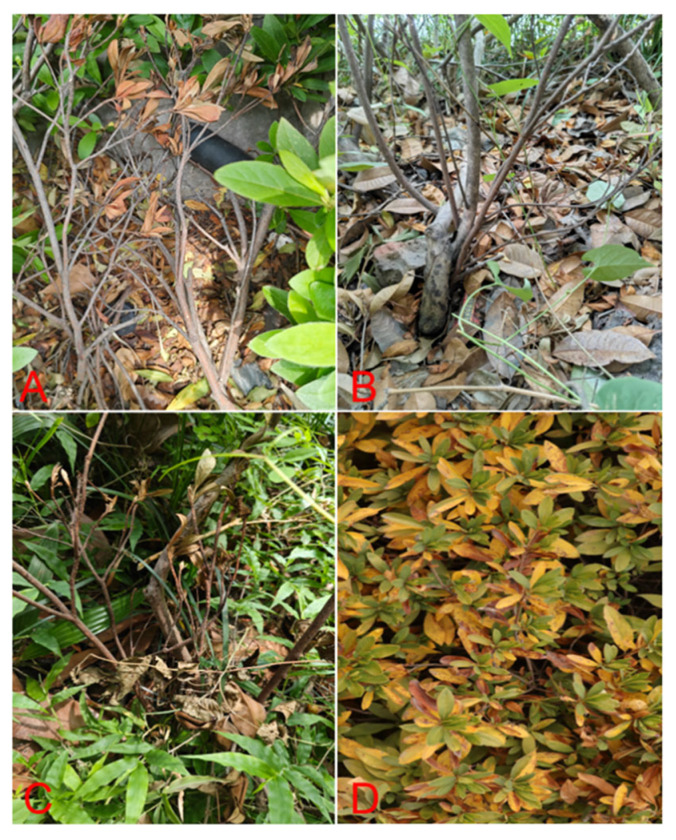
Field infection symptoms of *R. simsii*. (**A**) The young leaves at the top show signs of withering. (**B**) Brown spots with water stains appear on the roots. (**C**) The branches and leaves gradually withered and shriveled. (**D**) Brown spots form on the leaves and eventually spread across the entire leaf, causing it to turn yellow.

The disease affects approximately 15% of *R. simsii* plants in Nanjing from 2021 to 2023 and is characterized by crown and root rot. The top young leaves initially appeared dry. Symptoms spread from top to bottom, with branches and leaves wilting and drying out, and brown spots forming on the leaves, eventually spreading to the entire leaf. In addition, there are also symptoms of brown spots with water spots on the roots, rot and peeling, xylem became dark brown (Figure 1).

### 3.2. Pathogenicity of Fungal Isolates

**Figure 2:**
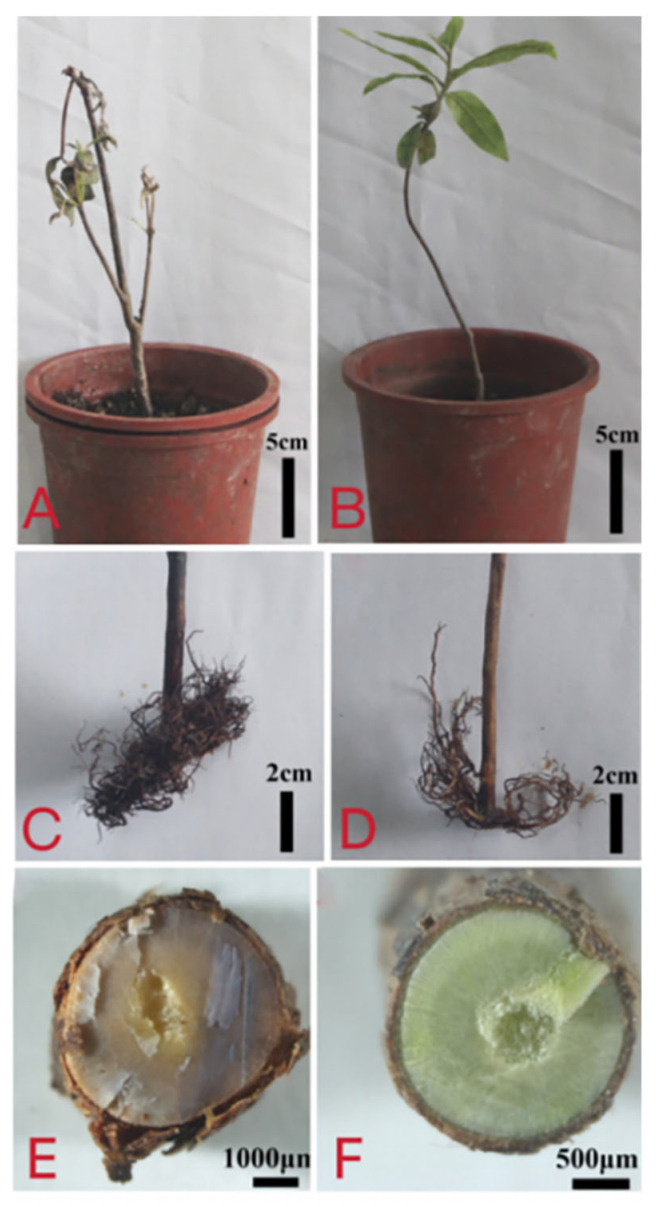
Pathogenicity of *P. vexans* on the roots of *R. simsii* inoculated artificially with zoospores. (**A**) Symptoms of *R. simsii* after 21 inoculation of roots with zoospore suspension of *P. vexans*. (**B**) Control of *R. simsii* symptoms. (**C**) Root rot symptoms after inoculation with *P. vexans*. (**D**) Control group root. (**E**) Cross-section of stem base of diseased plant inoculated with spore suspension of *P. vexans*. (**F**) Healthy stem base cross-section of the control group.

A large number of strains were isolated and purified from the infected roots and classified based on colony morphology. Among them, three strains with consistent colony morphology, mycelial color and mycelial growth rate were named DT10, DT15 and DT16. DT10 was selected from the three strains for two-year pot experiments on *R. simsii*. The spore suspension inoculation method was adopted to inoculate the healthy roots of *R. simsii*. Seven days after inoculation, the base of the stem turned black, the leaves began to droop but did not fall off, and wilting symptoms appeared. Twenty-one days after inoculation, the leaves of *R. simsii* withered and drooped, and brownish spots appeared. All inoculated seedlings (*n* = 9) showed root rot symptoms identical to those of naturally infected plants (Figure 2A,C,E). In comparison, control seedlings (*n* = 5) remained asymptomatic (Figure 2B,D,F). The pathogen was successfully reisolated from all inoculated plants. This experiment was repeated three times to confirm the results. The whole plant died. After the roots were dug out, it was found that the root tissue turned black and rotted, and water-soaked spots appeared. The results showed that all the above strains could cause the whole plant of *R. simsii* to be diseased, while the control group did not get sick. Moreover, the disease symptoms of *R. simsii* caused by DT10 were consistent with the natural disease symptoms in the wild. The inoculated diseased roots were re-isolated, and the morphology of the re-isolated strains was completely consistent with that of the inoculated strains. The control group did not get sick, and no strains were isolated.

### 3.3. Morphological Characteristics of the Pathogens

**Figure 3:**
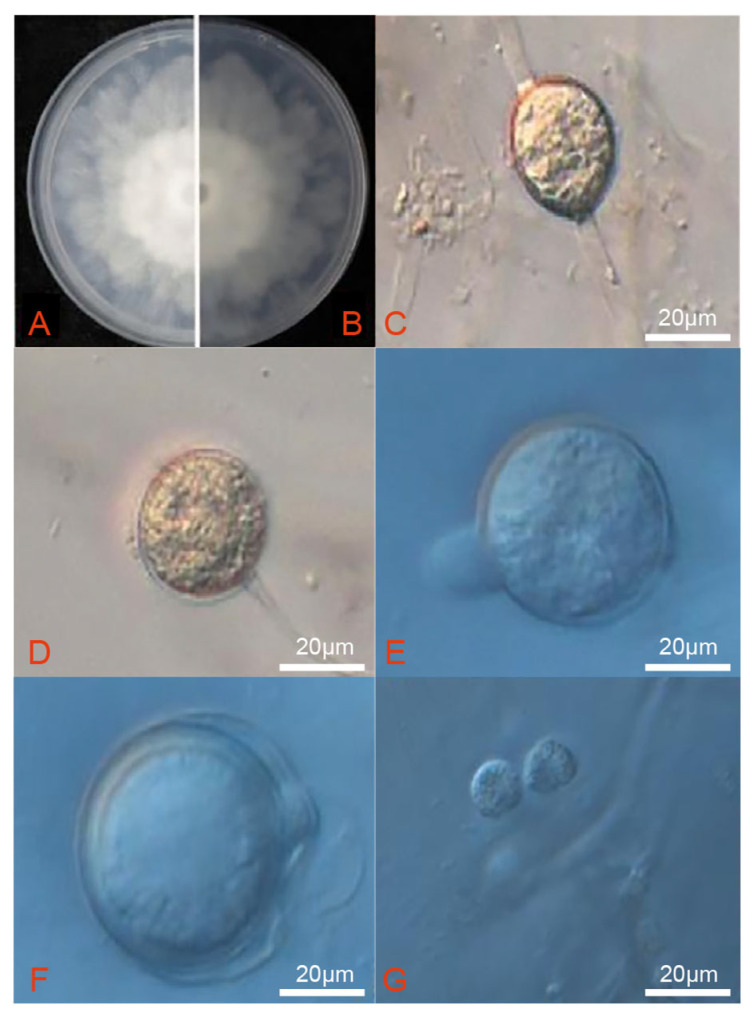
Morphological characters of *P. vexans*. (**A**,**B**) Colony morphology of three-day-old isolate DT10 grown on PDA. (**C**) Hyphae swelling. (**D**) The terminal hyphal hypha was swelling. (**E**) Sporangia show protuberances. (**F**) Oospore. (**G**) Zoospores.

The V8 juice was replaced with 10 mL of sterile water and 3 drops of unsterilized soil extract solution to stimulate the formation of sporangia and zoospores. After being cultivated on V8 medium for three days, strain DT10 presented a radial colony with sparse aerial mycelium (Figure 3A,B). Intercalary and terminal hyphae swelled and had dimensions of 19.7 ± 2.3 µm length × 18.3 ± 2.1 µm. Sporangia were spherical, pear-shaped, or ovoid with a papilla and measured between 23.1 and 28.6 µm (mean 25.7 µm) in length × 22.6 to 27.4 µm (mean 24.8 µm) in width (Figure 3C,D). Oospores were located mainly at the ends of short lateral branches, occasionally positioned laterally or internally, and exhibited a spherical shape with dimensions of 23.42 ± 1.7 µm in length and 20.6 ± 1.3 µm in width (Figure 3E,F). Zoospores had a diameter of 6.7 to 10.3 µm (Figure 3G).

### 3.4. Phylogenetic Analyses of the Pathogens

Genomic DNA extracted from three isolates was amplified using three different genetic markers. The resulting fragments were separated by gel electrophoresis, and the bands observed corresponded to the expected sizes. These amplified sequences were then subjected to BLAST 2.16.0 alignment analysis in the NCBI database. The results are shown in Table 3. Table 4 shows the GenBank accession numbers for the sequenced *Phytophthora* spp. Three gene fragments of LSU, COXI, and COXII were amplified by PCR. Primers amplified the LSU gene sequence with NL1/NL4, the COXI gene sequence with OomCoxI-Levup/FM85mod, and the COXII gene sequence with FM58/FM66. The results showed that the LSU, COXI, and COXII sequences ninnwgof strain DT10 (GenBank no. ON627804.1, ON623508.1, ON623510.1) and strain FBG2017010 (*P. vexans*, the sequence homology of LSU, COXI, and COXII genes of MT533451.1, FBG20181 (MT076052.1), and STE-U6741 (GU133560.1) were 100% and 99.20%, and 99.49%. Using P. multibullata as an outgroup, a phylogenetic tree was constructed by connecting LSU, COXI, and COXII gene fragments. The results showed that strains DT10, DT15, and DT16 belonged to the same clade. Strains DT10, DT15, and DT16 were confirmed to be *P. vexans* based on their grouping within the same branch with 100% affinity, confirming their identification as *P. vexans*.

**Figure 4:**
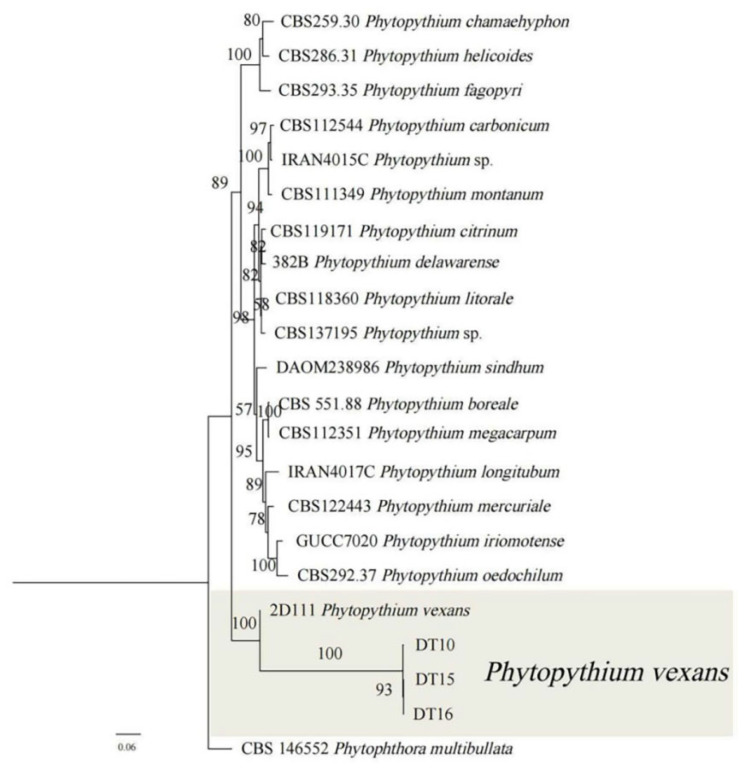
Maximum Likelihood analyses of *Phytopythium* species were constructed using the concatenated dataset (LSU, COXI and COXII). *P. vexans* (DT10, DT15 and DT16) in this study formed a monophyletic clade with other isolates of the same species. Bootstrap support values (ML ≥ 50) were shown at the nodes. The scale bar shows the predicted number of substitutions per nucleotide position. *Phytopythium capsici* was used as an outgroup.

The results of maximum likelihood analysis (ML) and Bayesian inference analysis (Bl) revealed a largely congruent phylogenetic tree, suggesting that the inferred evolutionary relationships between the Phytopythium isolates were statistically robust. A consensus tree was constructed using the RAxML bootstrap proportions (BP) derived from the ML analysis (Figure 4). Phylogenetic analysis revealed that isolates DT10, DT15, and DT16 formed a cluster on the branch associated with isolate 2D111, which had bootstrap support of 93%. The isolates were determined to be *P. vexans* on the basis of their morphological features and pathogenicity tests. The morphology of pathogens, including characteristics such as spore structure and hyphal features, can be influenced by culture conditions such as temperature and the composition of the culture medium, potentially leading to subjective misinterpretations. To overcome the limitations associated with morphological identification—namely its susceptibility to environmental factors and observer bias—and to enable objective and quantifiable analysis of pathogens, we performed molecular identification based on stable genetic markers, such as the LSU, COXI, and COXII gene sequences. This approach effectively addresses the shortcomings of traditional morphological methods and establishes a foundation for subsequent research on precise detection and control strategies.

### 3.5. Screening of Endophytic Bacteria for Biocontrol

**Figure 5:**
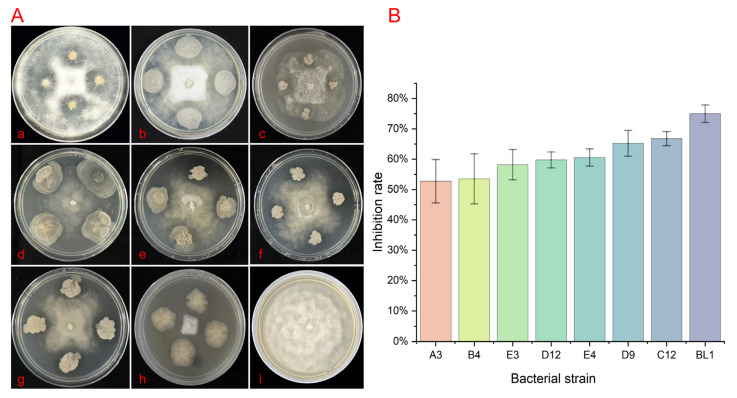
Screening results of endophytic bacteria for biological defense. (**A**) Inhibition of mycelial growth of *P. vexans* by endophytic bacteria using the four-point plate stand of method. (**a**–**h**) correspond to A3, B4, E3, D12, E4, D9, C12, and BL1, which were isolated from healthy soil, and (**i**) represents the Blank group. (**B**) Inhibition rate of pathogens by endophytic bacteria in the biological defense experiment.

In the plate confrontation experiment, when the colonies of the pathogen (target bacteria) and the tested antagonistic bacteria came into contact at their interface, phenomena such as hyphal lysis and colony collapse were observed in the overlapping region. The growth of the pathogen was inhibited, resulting in the formation of a distinct transparent inhibition zone. one exhibited the most pronounced antagonistic effect and was selected for further analysis as shown in Figure 5(A,h). The colonies of the antagonistic strain BL1 on LB plates are irregularly circular, white, with wrinkles on the surface, not smooth, and have obvious protrusions at the top. Later, obvious serrated wrinkles appear at the edges. On the V8 medium, the inhibition rates of A3, B4, E3, D12, E4, D9, C12, and H3 were 52.73%, 53.52%, 58.20%, 59.77%, 60.55%, 65.23%, 66.80%, and 75.00%, respectively (Figure 5B). These in vitro results demonstrate the direct antagonistic potential of strain BL1 against *P. vexans*. It is important to note that this activity was observed under controlled laboratory conditions, and its efficacy in plant or soil environments remains to be determined. The comparison results of BL1 are presented in Supplementary Result S1; The comparison results of the inhibition rates are presented in Result S2.

### 3.6. Whole-Genome Sequencing of Antagonistic Bacteria

**Figure 6:**
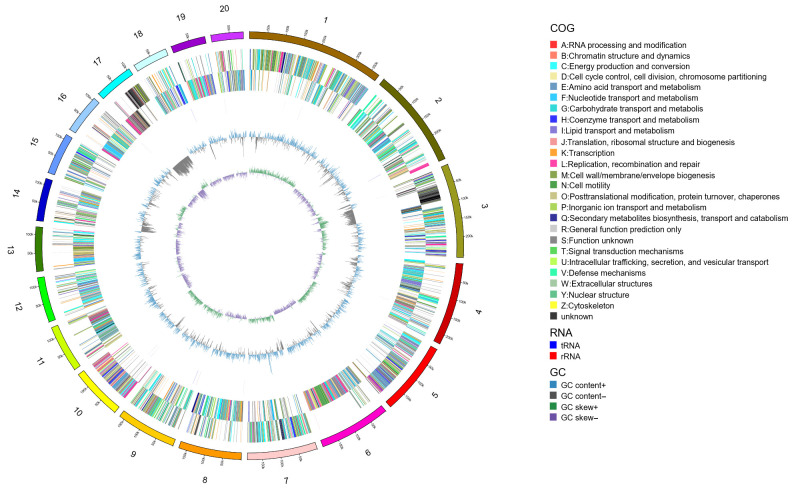
Circular representation of Bacillus licheniformis BL1 genome (from the outside in, they are the karyotype of the chromosome, CDS on the positive and negative strands (different colors indicate different COG (Clusters of Orthologous Groups) classifications of CDS (Coding Sequences)), transfer RNA and ribosomal RNA, GC content, and the innermost circle is GC-skew).

**Figure 7:**
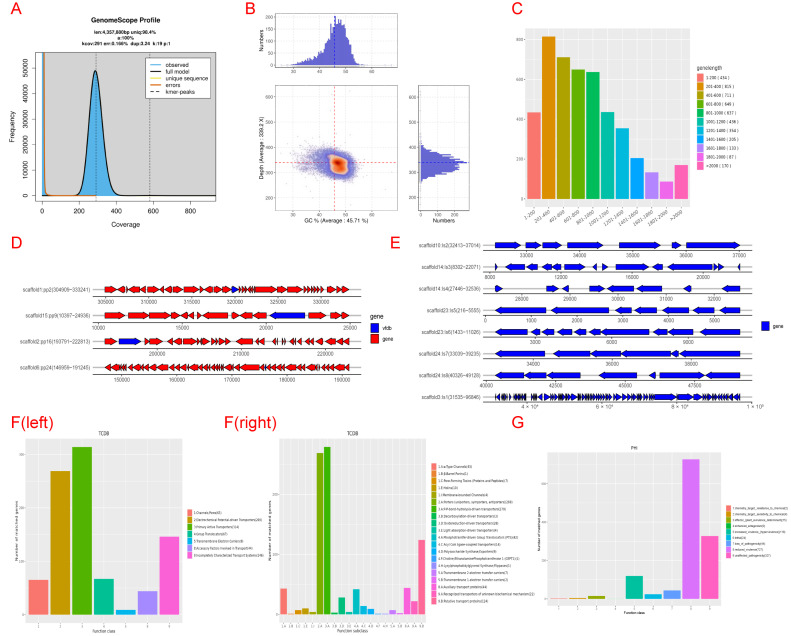
The whole-genome sequencing results of Bacillus licheniformis BL1 bacteria. (**A**) 19-kmer distribution elliptic plot. (**B**) Distribution map of GC_Depth. (**C**) Gene length distribution map. (**D**) The distribution map of cards and vfs on the prophage. (**E**) Gene island gene distribution map. (**F**) To the left of F is the first-level classification diagram of TCDB functions, and to the right of F is the second-level classification diagram of TCDB functions. (**G**) Distribution map of PHI phenotypic mutation types.

After analyzing the scaffold assembled by BL1 using the checkM v1 software, the completeness was 98.96. Using the circos v0.69-3 software, the first 20 scaffolds with sequence lengths greater than 5000 bp in the sample were plotted, and the results are shown in Figure 6. The distribution of K-mer was calculated using JELLYFISH 2.3.0 software. kmer = 19 was selected. The depth distribution of K-mer is shown in Figure 7A. According to the genomic evaluation results, the genome size of BL1 is 4,357,880 bp. The distribution of GC content and sequencing depth of the genome is shown in Figure 7B.

### 3.7. Genomic Component Analysis and the Coding Gene Annotation

Genomic analysis of strain BL1 (Figure 7) reveals several key features: protein-coding genes were predicted using GeneMarkS (length distribution in Figure 7C). Prophage screening (PhiSpy) detected virulence factors (e.g., card toxin genes) on prophages (Figure 7D,E), which unexpectedly enhance antibacterial secretion despite carrying pathogenic elements. Transporter classification followed TCDB standards (Figure 7F). Pathogen–host interaction (PHI) analysis identified genes affecting virulence regulation (Figure 7G), with most genes reducing pathogenicity (327 genes). Fewer genes caused lethality (24 genes), acted as effectors (15 genes), or completely eliminated pathogenicity (44 genes). Crucially, the genome encodes quorum-quenching enzymes like AiiA lactonase, which breaks down communication molecules (AHLs) of pathogenic *P. vexans*. This blocks the pathogen’s virulence gene expression and enzyme secretion.

**Figure 8:**
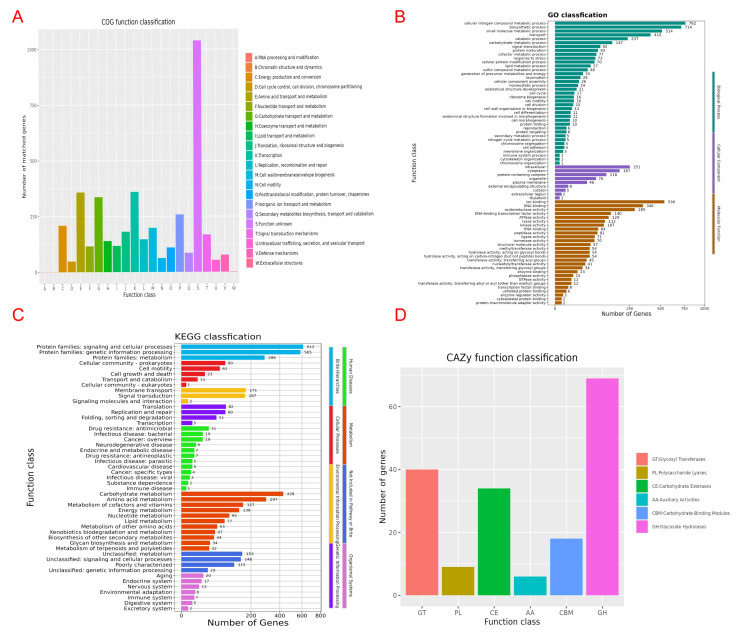
Coding gene annotation results of *Bacillus licheniformis* BL1 bacteria. (**A**) COG function classification. (**B**) GO classification. (**C**) KEGG classification. (**D**) CAZy function classification.

Functional annotation of strain BL1 (Figure 8) reveals key genomic characteristics. eggNOG classification shows the highest number of genes with unknown function (Class S, >1000), followed by genes for translation/ribosome function (Class J) and energy metabolism (Class C). GO enrichment highlights abundant genes involved in biological processes—notably cellular nitrogen compound metabolism—and molecular functions like ion binding and transport. KEGG pathway analysis further confirms substantial gene representation in signal transduction and cellular processes (612 genes), genetic information processing (585 genes), and core metabolic pathways: carbohydrate metabolism (428 genes), amino acid metabolism (297 genes), energy metabolism (138 genes), and lipid metabolism (77 genes). CAZy database annotation identifies particularly high counts of glycoside hydrolase (GH, >69) and glycosyltransferase (GT, ~40) genes. Collectively, these results demonstrate that BL1 possesses highly developed systems for genetic information processing and carbohydrate metabolism, robust signal transduction capabilities with expanded regulatory functions, and essential modules for core metabolism and environmental adaptation, alongside a significant number of uncharacterized genes.

## 4. Discussion

The pathogen that causes root rot can infect not only *R. simsii*, but also other potential host plants [44]. Therefore, it is essential to identify the pathogen causing root rot of *R. simsii* in time. On the basis of morphology, molecular and phylogenetic analysis, *P. vexans* was identified as the causal crown and root rot agent on *R. simsii* in China. To our knowledge, there have been no reports of *P. vexans* causing crown and roosting on *R. simsii* [44].

This new record adds *R. simsii* to the expanding host range of *P. vexans* of a trend observed globally with woody and ornamental plants. For instance, similar to our findings on rhododendron, *P. vexans* was recently identified as the cause of a destructive root rot on eastern redbud (*Cercis canadensis*) in Tennessee, USA, marking its first report on this host and in the region [45]. The pathogen’s capacity to emerge in new geographical areas and attack previously unreported woody hosts. Furthermore, the threat from related oomycetes is also increasing. A 2022 review highlighted the emergence of numerous Phytophthora species in natural and managed ecosystems worldwide, often facilitated by the global plant trade, leading to significant ecological and economic damage [46]. Our finding of *P. vexans* in a Chinese nursery aligns with this pattern of human-mediated pathogen spread. The epidemiological profile of *P. vexans* shares key traits with other high-impact oomycetes. Like many Phytophthora species, it is a soil- and water-borne pathogen, and its management in nursery settings is notoriously challenging. A study on Phytophthora root rot in apple orchards demonstrated how these pathogens can persist and spread through irrigation water, a risk that is equally relevant for the cultivation of *R. simsii* in China [47]. The symptoms we observed—root necrosis, wilting, and plant death—are classic manifestations of oomycete infections, reflecting a conserved strategy of host colonization that disrupts water and nutrient uptake.

Due to the widespread cultivation of rhododendron, several diseases have affected its economic, ornamental, and medicinal value, leading to its decline. Three new species of plant pathogenic fungi were discovered in Yunnan and Jiangxi provinces of China. They, respectively, infect rice flower (*Lyonia ovalifolia*) and two species of rhododendrons (*R. simsii* and *R. russatum*), belonging to the typical leaf-swelling pathogen genus Exbasidium [48]. This discovery is of reference value for the study of regional plant disease ecology and the prevention and control of rhododendron diseases [49]. *Pseudocercospora rhododendricola* caused leaf dark spots in *Rhododendron* spp. in Zhanjiang, China [44]. Foliage blight and shoot dieback caused by *Phytophthora pini* was first reported in *Rhododendron* spp. in Nanjing, China [50]. *Phytophthora vexans*, a globally widespread pathogen that can cause crown rot, root rot, stem rot, and leaf blight, affects a wide array of woody plant species. Academic research has consistently shown that this pathogen primarily targets woody plants and key economic crops. For instance, *P. vexans* causes root rot in China, leads to collar rot in kiwifruit orchards in Turkey [25], triggers stem rot on avocado trees in Mexico, and results in wilting in apple orchards in Morocco [51]. In addition, in Korea, the pathogen was isolated from *Anthurium andraeanum*, which was infected [52]. The severity and diversity of diseases caused by *P. vexans* and its relatives highlight a significant global challenge. A meta-analysis of Phytophthora diversity in forests and nurseries across Europe revealed a high prevalence of cryptic species and hybrids, complicating detection and management efforts [49]. Notably, in 2023, *P. vexans* was identified for the first time as the causal agent of root rot in redbud trees in Tennessee, United States [53]. In summary, the widespread ecological and economic damage caused by *P. vexans* on a global scale emphasizes the urgent need to implement rigorous management approaches to curb its spread and mitigate its far-reaching impacts. This disease poses a critical menace to the cultivation of *R. simsii* because it has the potential to culminate in the plant’s demise, markedly reducing its esthetic and monetary worth. The severity of the disease warrants heightened societal awareness, necessitating the prompt development of appropriate preventive and management strategies. These measures curb the disease’s proliferation, minimize its impact on host plants, and serve as a foundation for further research into its emergence.

Data on the isolation and characterization of pathogenic samples were collected through a comprehensive survey conducted in Nanjing. Notably, *P. vexans* was isolated from 65% of the samples collected. With the increasing number of reports of diseases caused by *P. vexans*, the cultivation of *R. simsii* faces considerable risks. Therefore, it is important to focus on this issue and to develop and formulate effective measures to mitigate the adverse effects of this disease. As an important ornamental plant, *R. simsii* is widely planted in China. The isolation and identification of these pathogens will provide a new approach to the cultivation and management of *R. simsii* in China. Regarding crown and root rot on *R. simsii*, there are a lot of problems to be solved. *P. vexans* is likely to be a major environmental threat in the future, as the pathogen can infect a wider range of hosts. It is recommended to test the soil in and around areas where rhododendron Crown and Root Rot occur, and to make appropriate use of fungicides and fertilizers, so as to maintain proper environmental conditions and prevent the occurrence of the disease [54].

*B. licheniformis* strain BL1 emerges as a promising biocontrol agent (BCA) against *P. vexans*, and our genomic analysis provides compelling mechanistic insights into its antagonistic potential. The BL1 genome (4.31 Mb, GC 45.73%) revealed key functional enrichments directly relevant to its observed inhibitory activity. Gene annotation identified significant enrichment in pathways for secondary metabolite synthesis, particularly lipopeptide antibiotics. Crucially, we detected genes encoding non-ribosomal peptide synthetases (NRPSs) and polyketide synthases (PKSs), the core enzymatic machinery responsible for producing diverse antimicrobial lipopeptides like surfactin, iturin, and fengycin in *Bacillus* spp. [55,56,57,58]. These lipopeptides are well documented for their ability to disrupt fungal and oomycete membrane integrity, leading to cell lysis and death [58]. The presence of these biosynthetic gene clusters provides a direct molecular basis for BL1′s observed antagonism and suggests the production of potent membrane-targeting compounds against *P. vexans*. Analysis using the CAZy database was particularly revealing. We found a high abundance of genes encoding Glycoside Hydrolases (GHs). Significantly, several of these GHs belong to families known to target β-1,3-glucan (e.g., GH16, GH17, GH55, GH64, GH81) and cellulose (e.g., GH5, GH6, GH9, GH12, GH44, GH45, GH48, GH74, GH124), which constitute the primary structural components (>90%) of the *P. vexans* cell wall. This abundance contrasts sharply with the minimal representation of chitinase genes (e.g., GH18, GH19), consistent with the negligible chitin content in oomycetes. The specific enrichment of β-1,3-glucanases and cellulases in BL1 provides a clear genomic explanation for its effectiveness: these enzymes directly hydrolyze the main structural polysaccharides of *P. vexans*, leading to cell wall weakening, protoplast leakage, osmotic imbalance, and ultimately, inhibition of hyphal growth and integrity. This aligns perfectly with the observation that BL1′s antagonism primarily targets the mycelial growth stage, where active cell wall synthesis and extension occur. The genomic evidence for biosynthetic gene clusters of antimicrobial lipopeptides and a suite of cell wall-degrading enzymes suggests a potential dual-action mechanism that could explain the observed in vitro antagonism [59]. Genomic analysis [59] further revealed genes associated with Type II (Sec pathway) and Type VI (T6SS) secretion systems. These systems are critical for the efficient extracellular delivery of the identified antimicrobial compounds (lipopeptides, enzymes) and potentially other effector molecules into the environment or directly at the pathogen interface [60]. The enrichment of genes related to “membrane transport” (COG Class E, TCDB analysis) and “signal transduction” (COG Class T) supports the notion that BL1 possesses sophisticated mechanisms for nutrient uptake and environmental sensing/response. This capability allows it to competitively acquire nutrients in the rhizosphere niche and potentially interfere with pathogen signaling, further restricting *P. vexans* growth and virulence expression [61]. The predicted presence of quorum quenching enzymes (e.g., AiiA lactonase) strengthens this hypothesis. The BL1 genome also encodes numerous genes involved in stress response (COG Class V) and osmoprotectant synthesis/transport. These include systems for managing oxidative stress (e.g., catalases, peroxidases), osmotic stress (e.g., compatible solute transporters/synthesizers like proline/betaine), and potentially nutrient limitation [62]. This genomic endowment likely enhances BL1′s fitness and persistence in the challenging plant rhizosphere and soil environments, enabling it to maintain its antagonistic activity under varying conditions and compete effectively with *P. vexans* and other microbes. While a full comparative genomic analysis is beyond the scope here, preliminary assessment indicates that BL1 possesses a robust repertoire of the core Bacillus biocontrol genes (NRPS/PKS, key GHs, secretion systems). Its specific complement of GH genes, heavily skewed towards cellulase activity, appears particularly well adapted for targeting oomycete pathogens like *P. vexans*, compared to strains primarily effective against true fungi (which rely more on chitinases). Furthermore, the absence of known major virulence factors typical of plant-pathogenic bacteria in the annotated genome reinforces its safety profile as a potential BCA.

A primary limitation of this study is that the biocontrol potential of BL1 is currently supported only by in vitro evidence. While the plate confrontation assay and genomic analysis provide a strong foundation and compelling hypotheses for its mode of action, the complex rhizosphere environment and plant–pathogen-bacterium interactions can significantly alter efficacy. The promising in vitro activity and genetic capacity of BL1 must now be validated through in planta experiments. Future work will focus on evaluating the protective effect of BL1 on *R. simsii* seedlings under greenhouse and eventually field conditions, to confirm its ability to reduce disease severity and promote plant growth in the presence of *P. vexans*. In summary, the genome of *B. licheniformis* BL1 paints a coherent picture of a strain equipped for effective.

## 5. Conclusions

In June 2021–2023, a new disease of *R. simsii* was observed in a nursery in Xuanwu Lake, Nanjing. Dozens of seriously diseased *R. simsii* roots were excavated. The entire plant exhibited signs of wilting, with the leaves turning yellow and eventually culminating in complete withering. The study elucidated the etiology of the disease affecting *R. simsii*. Disease causation was determined through initial whole-plant symptom observation, followed by isolation of the infected tissue and identification of the pathogen based on the morphology of sporangia, oospores, and zoospores. Through a combination of pathogenicity tests, and multi-gene phylogenetic analysis (LSU, COXI, COXII), the causal agent was identified as *P. vexans*. These findings will provide a benchmark for further research into diseases that affect *R. simsii*. This is the first report of *P. vexans* causing this disease on *R. simsii* in China.

*Bacillus* spp. is a widely distributed microorganism that readily forms dominant microbial populations on the surfaces and in the rhizospheres of soils and plants. Researchers have focused on isolating and identifying Bacillus strains with high antagonistic activity against various plant pathogens. To achieve this objective, an endophytic bacterial strain, BL1, was isolated from the rhizosphere soil of healthy plants and identified as Bacillus licheniformis. In vitro plate confrontation assays demonstrated that BL1 has strong antagonistic activity against *P. vexans*. Whole-genome sequencing and genomic analysis of BL1 revealed the presence of gene clusters involved in the synthesis of antimicrobial lipopeptides and a suite of cell wall-degrading enzymes (e.g., glycoside hydrolases), providing a genetic basis for its observed inhibitory effects. This study also found that the oomycete inhibitory effect of strain BL1 on *P. vexans* was mainly targeted at the mycelial growth stage. This phenomenon may result from the fact that the target of oomycete inhibitory substances is more sensitive at the mycelial stage, or there are significant differences in the metabolic characteristics of pathogenic oomycetes at different growth stages. It is important to note that these biocontrol findings are preliminary and derived from laboratory assays. Therefore, particularly in planta experiments is essential to confirm the efficacy of BL1 in controlling *P. vexans* infections in living *R. simsii* plants and to evaluate its potential for practical application in further research.

According to the concept of “preventive biological control”, it is recommended to apply it during the active period of pathogen mycelium to achieve the best control effect. Further, it is recommended to use it in combination with biological control agents of other mechanisms of action, so as to construct a more comprehensive and efficient control system to deal with *P. vexans*.

## Figures and Tables

**Table 1:** BLAST 2.16.0 results are obtained from the LSU, COXI, and COXII of three representative.

Blast Match Sequence				Sequence Identity (%)
Isolate	DNA Target	GenBank Accession Numbers	Reference Accession No.	
DT10	LSU	ON627804.1	*P. vexans* FBG2017010 (MT533451.1)	100%
	COXI	ON623508.1	*P. vexans* FBG20181 (MT076052.1)	99.20%
	COXII	ON623510.1	*P. vexans* STE-U6741 (GU133560.1)	99.49%
DT15	LSU	OQ754108	*P. vexans* FBG2017010 (MT533451.1)	100%
	COXI	ON623509.1	*P. vexans* STE-U6708 (GU133476.1)	98.81%
	COXII	ON623511.1	*P. vexans* STE-U6741 (GU133560.1)	99.83%
DT16	LSU	MZ519893	*P. vexans* KACC:410989 (PV473534.1)	100%
	COXI	OQ753101	*P. vexans* CBS119.80 (CBS119.80)	99.32%
	COXII	OQ753097	*P. vexans* STE-U6741 (GU133560.1)	99.49%

Sequence Identity (%) indicates the percentage of identical nucleotide matches between the query sequence (our isolate) and the top hit reference sequence in the GenBank database using the BLASTn algorithm.

**Table 2:** NCBI Accession Numbers for Phylogenetic Studies.

Phytophthora Species	GenBank Accession Numbers			
	Isolate	LSU	COXI	COXII
*P. vexans*	DT10	ON627804.1	ON623508.1	ON623510.1
*P. vexans*	DT15	OQ754108	ON623509	ON623511
*P. vexans*	DT16	MZ519893	OQ753101	OQ753097
*P. vexans*	2D111	AB856796.1	AB856784.1	AB948193.1
*Phytopythium* sp.	CBS137195	AB948194.1	AB948191.1	AB948191.1
*Phytopythium* sp.	IRAN40150	MT729975.1	MT720654.1	MT720670.1
*Ph. boreale*	CBS 551.88	HO665261.1	EF408872.1	EF408876.1
*Ph. chamaehyphon*	CBS259.30	AB690593.1	AB690644.1	AB690674.1
*Ph. citrinum*	CBS119171	AB690597.1	AB690649.1	AB690679.1
*P. helicoides*	CBS286.31	AB690594.1	AB690645.1	AB690675.1
*Ph.chamaehyphon*	CBS259.30	AB690593.1	AB690644.1	AB690674.1
*Ph. iriomotense*	GUCC7020	AB690607.1	AB690659.1	AB690689.1
*Ph. sindhum*	DAOM238986	HO643396.2	HO708443.1	
*Ph. mercuriale*	CBS122443	AB690585.1	AB690636.1	AB690666.1

Isolate refers to the strain designations used in this study. All reference sequences were downloaded from the NCBI GenBank database (https://www.ncbi.nlm.nih.gov/genbank/ accessed on 26 March 2025).

**Table 3:** List of PCR amplification primers in this article.

Gene	Primer	Sequence (5′–3′)	PCR Conditions
LUS	NL1	ACCCGCTGAACTYAAGC	94 °C, 3 min; (94 °C, 30 s; 58 °C, 30 s; 72 °C, 45 s) × 35; 72 °C, 10 min
	NL4	CGCCAGACGAGCTTACC	
COXI	OomCoxI-Levup	TCAWCWMGATGGCTTTTTTCAAC	94 °C, 3 min; (94 °C, 30 s; 56 °C, 30 s; 72 °C, 45 s) × 35; 72 °C, 10 min
	FM85mod	RRHWACKTGACTDATRATACAAA	
COXII	FM58	GTATTTCTTCTTTATTAGGTGC	94 °C, 3 min; (94 °C, 30 s; 56 °C, 30 s; 72 °C, 45 s) × 35; 72 °C, 10 min
	FM66	CGTGAACTAATGTTACATATAC	

LSU, Large subunit ribosomal RNA gene; COXI, Cytochrome c oxidase subunit I gene; COXII, Cytochrome c oxidase subunit II gene. PCR conditions follow a standard three-step amplification process.

**Table 4:** Primer sequences and PCR amplification procedures used for the identification of endophytic bacteria.

Locus	Primer	Sequence (5′–3′)	PCR Conditions
16S ribosomal DNA (16S rDNA)	27F	AGAGTTTGATCCTGGCTCAG	94 °C, 3 min; (94 °C, 30 s, 58 °C, 30 s; 72 °C, 15 s) × 30; 72 °C, 10 min
	1492R	TACGGYTACCTTGTTACGACTT	

## Data Availability

The original contributions presented in this study are included in the article/Supplementary Material. Further inquiries can be directed to the corresponding author.

## Supplementary Materials

The following supporting information can be downloaded at: https://www.mdpi.com/article/10.3390/microorganisms13112417/s1, Result S1: The comparison results of BL1, Result S2: The comparison results of the inhibition rates.
