# Supplementary material for: Incidence of Crown and Root Rot in Rhododendron simsii Caused by Phytopythium vexans in China and Screening of Endophytic Bacteria for Biocontrol
Source: Microorganisms. 2025 Oct 22;13(11):2417. doi: 10.3390/microorganisms13112417 (PMC12654715; doi:10.3390/microorganisms13112417)
Supplement: Supplementary file 1 [file microorganisms-13-02417-s001.zip › Result S1.pdf]

BLAST ® » blastn suite » results for RID-Z967BC4E016

Job Title	32025040700118_(Z7497) ...
RID	Z967BC4E016 Search expires on 04-09 11:32 am
Program	BLASTN
Database	rRNA_typestrains/16S_ribosomal_RNA
Query ID	lcl|Query_6057875
Description	None ...
Molecule type	dna
Query Length	1409

Descriptions

Description	Scientific Name	Max Score	Total Score	Query Cover	E value	Per. Ident	Acc. Len	Accession
Bacillus licheniformis strain BCRC 11702 16S ribosomal RNA, partial sequence	Bacillus licheniformis	2553	2553	100%	0.0	99.43%	1468	NR_116023.1
Bacillus licheniformis strain DSM 13 16S ribosomal RNA, partial sequence	Bacillus licheniformis	2553	2553	100%	0.0	99.43%	1545	NR_118996.1
Bacillus licheniformis strain NBRC 12200 16S ribosomal RNA, partial sequence	Bacillus licheniformis	2547	2547	100%	0.0	99.29%	1475	NR_113588.1
Bacillus licheniformis strain ATCC 14580 16S ribosomal RNA, partial sequence	Bacillus licheniformis	2542	2542	100%	0.0	99.29%	1545	NR_074923.1
Bacillus haynesii strain NRRL B-41327 16S ribosomal RNA, partial sequence	Bacillus haynesii	2536	2536	100%	0.0	99.22%	1508	NR_157609.1
Bacillus sonorensis strain NBRC 101234 16S ribosomal RNA, partial sequence	Bacillus sonorensis	2525	2525	100%	0.0	99.08%	1475	NR_113993.1
Bacillus aerius strain 24K 16S ribosomal RNA, partial sequence	Bacillus aerius	2508	2508	100%	0.0	98.87%	1494	NR_042338.1
Bacillus sonorensis strain NRRL B-23154 16S ribosomal RNA, partial sequence	Bacillus sonorensis	2507	2507	99%	0.0	99.00%	1410	NR_025130.1
Bacillus swezeyi strain NRRL B-41294 16S ribosomal RNA, partial sequence	Bacillus swezeyi	2473	2473	100%	0.0	98.44%	1507	NR_157608.1
Bacillus amyloliquefaciens strain BCRC 11601 16S ribosomal RNA, partial sequence	Bacillus amyloliquefaciens	2435	2435	100%	0.0	97.94%	1468	NR_116022.1
Bacillus nakamurai strain NRRL B-41091 16S ribosomal RNA, partial sequence	Bacillus nakamurai	2435	2435	100%	0.0	97.94%	1508	NR_151
Bacillus atrophaeus strain NBRC 15539 16S ribosomal RNA, partial sequence	Bacillus atrophaeus	2431	2431	100%	0.0	97.87%	1475	NR_112723.1

Description	Scientific Name	Max Score	Total Score	Query Cover	E value	Per. Ident	Acc. Len	Accession
Bacillus amyloliquefaciens strain NBRC 15535 16S ribosomal RNA, partial sequence	Bacillus amyloliquefaciens	2431	2431	100%	0.0	97.87%	1475	NR_112685.1
Bacillus amyloliquefaciens strain NBRC 15535 16S ribosomal RNA, partial sequence	Bacillus amyloliquefaciens	2429	2429	100%	0.0	97.87%	1472	NR_041455.1
Bacillus atrophaeus strain JCM 9070 16S ribosomal RNA, partial sequence	Bacillus atrophaeus	2429	2429	100%	0.0	97.87%	1515	NR_024689.1
Bacillus amyloliquefaciens strain MPA 1034 16S ribosomal RNA, partial sequence	Bacillus amyloliquefaciens	2429	2429	100%	0.0	97.87%	1448	NR_117946.1
Bacillus subtilis subsp. subtilis strain 168 16S ribosomal RNA, complete sequence	Bacillus subtilis subsp. subtilis	2423	2423	100%	0.0	97.80%	1550	NR_102783.2
Bacillus vallismortis strain NBRC 101236 16S ribosomal RNA, partial sequence	Bacillus vallismortis	2420	2420	100%	0.0	97.73%	1475	NR_113994.1
Bacillus inaquosorum strain BGSC 3A28 16S ribosomal RNA, partial sequence	Bacillus inaquosorum	2418	2418	100%	0.0	97.73%	1538	NR_104873.1
Bacillus stercoris strain D7XPN1 16S ribosomal RNA, partial sequence	Bacillus stercoris	2418	2418	100%	0.0	97.73%	1455	NR_181952.1
Bacillus stercoris strain JCM 30051 16S ribosomal RNA, partial sequence	Bacillus stercoris	2418	2418	100%	0.0	97.73%	1508	NR_180796.1
Bacillus cabrialesii strain TE3 16S ribosomal RNA, complete sequence	Bacillus cabrialesii	2418	2418	100%	0.0	97.73%	1550	NR_180419.1
Bacillus vallismortis strain DSM 11031 16S ribosomal RNA, partial sequence	Bacillus vallismortis	2418	2418	100%	0.0	97.73%	1530	NR_024696.1
Bacillus subtilis strain JCM 1465 16S ribosomal RNA, partial sequence	Bacillus subtilis	2412	2412	100%	0.0	97.66%	1472	NR_113265.1
Bacillus subtilis strain NBRC 13719 16S ribosomal RNA, partial sequence	Bacillus subtilis	2412	2412	100%	0.0	97.66%	1475	NR_112629.1
Bacillus spizizenii strain NBRC 101239 16S ribosomal RNA, partial sequence	Bacillus spizizenii	2412	2412	100%	0.0	97.66%	1475	NR_112686.1
Bacillus rugosus strain SPB7 16S ribosomal RNA, partial sequence	Bacillus rugosus	2412	2412	100%	0.0	97.66%	1548	NR_181236.1
Bacillus subtilis strain DSM 10 16S ribosomal RNA, partial sequence	Bacillus subtilis	2412	2412	100%	0.0	97.66%	1517	NR_027552.1
Bacillus tequilensis strain 10b 16S ribosomal RNA, partial sequence	Bacillus tequilensis	2412	2412	100%	0.0	97.65%	1456	NR_104919.1

Description	Scientific Name	Max Score	Total Score	Query Cover	E value	Per. Ident	Acc. Len	Accession
Bacillus velezensis strain CBMB205 16S ribosomal RNA, partial sequence	Bacillus velezensis	2410	2410	99%	0.0	97.79%	1445	NR_116240.1
Bacillus halotolerans strain LMG 22476 16S ribosomal RNA, partial sequence	Bacillus halotolerans	2407	2407	100%	0.0	97.59%	1468	NR_115929.1
Bacillus mojavensis strain NBRC 15718 16S ribosomal RNA, partial sequence	Bacillus mojavensis	2407	2407	100%	0.0	97.59%	1475	NR_112725.1
Bacillus halotolerans strain DSM 8802 16S ribosomal RNA, partial sequence	Bacillus halotolerans	2407	2407	100%	0.0	97.59%	1545	NR_115063.1
Bacillus subtilis strain BCRC 10255 16S ribosomal RNA, partial sequence	Bacillus subtilis	2407	2407	100%	0.0	97.59%	1468	NR_116017.1
Bacillus velezensis strain FZB42 16S ribosomal RNA, complete sequence	Bacillus velezensis	2407	2407	100%	0.0	97.59%	1550	NR_075005.2
Bacillus subtilis strain IAM 12118 16S ribosomal RNA, complete sequence	Bacillus subtilis	2407	2407	100%	0.0	97.59%	1550	NR_112116.2
Calidifontibacillus erzurumensis strain P2 16S ribosomal RNA, partial sequence	Calidifontibacillus erzurumensis	2407	2407	99%	0.0	97.92%	1401	NR_180225.1
Bacillus mojavensis strain IFO 15718 16S ribosomal RNA, partial sequence	Bacillus mojavensis	2407	2407	100%	0.0	97.59%	1526	NR_024693.1
Bacillus nematocida strain B-16 16S ribosomal RNA, partial sequence	Bacillus nematocida	2403	2403	100%	0.0	97.51%	1511	NR_115325.1
Bacillus halotolerans strain CR-95 16S ribosomal RNA, partial sequence	Bacillus halotolerans	2403	2403	100%	0.0	97.52%	1420	NR_115282.1
Bacillus siamensis KCTC 13613 strain PD-A10 16S ribosomal RNA, partial sequence	Bacillus siamensis KCTC 13613	2403	2403	100%	0.0	97.52%	1525	NR_117274.1
Calidifontibacillus erzurumensis strain P2 16S ribosomal RNA, partial sequence	Calidifontibacillus erzurumensis	2401	2401	99%	0.0	97.84%	1402	NR_178988.1
Bacillus spizizenii strain NRRL B-23049 16S ribosomal RNA, partial sequence	Bacillus spizizenii	2399	2399	99%	0.0	97.64%	1409	NR_024931.1
Bacillus mojavensis strain IFO 15718 16S ribosomal RNA, partial sequence	Bacillus mojavensis	2398	2398	99%	0.0	97.84%	1407	NR_118290.1
Bacillus rugosus strain SPB7 16S ribosomal RNA, partial sequence	Bacillus rugosus	2392	2392	98%	0.0	97.90%	1404	NR_180415.1
Bacillus licheniformis strain NCDO 1772 16S ribosomal RNA, partial sequence	Bacillus licheniformis	2375	2375	99%	0.0	96.50%	1429	NR_118959.1
Bacillus halotolerans strain CR-119 16S	Bacillus halotolerans	2359	2359	100%	0.0	96.89%	1514	NR_115283.1

Description	Scientific Name	Max Score	Total Score	Query Cover	E value	Per. Ident	Acc. Len	Accession
ribosomal RNA, partial sequence								
Bacillus subtilis strain NCDO 1769 16S ribosomal RNA, partial sequence	Bacillus subtilis	2351	2351	99%	0.0	97.00%	1427	NR_118972.1
Bacillus piscis strain 16MFT21 16S ribosomal RNA, partial sequence	Bacillus piscis	2344	2344	99%	0.0	97.06%	1474	NR_165685.1
Bacillus amyloliquefaciens DSM 7 = ATCC 23350 16S ribosomal RNA, partial sequence	Bacillus amyloliquefaciens DSM 7 = ATCC 23350	2335	2335	99%	0.0	96.57%	1427	NR_118950.1
Bacillus subtilis strain SBMP4 16S ribosomal RNA, partial sequence	Bacillus subtilis	2331	2331	99%	0.0	96.78%	1463	NR_118383.1
Bacillus altitudinis 41KF2b 16S ribosomal RNA, partial sequence	Bacillus altitudinis 41KF2b	2290	2290	100%	0.0	96.10%	1506	NR_042337.1
Bacillus stratosphericus strain 41KF2a 16S ribosomal RNA, partial sequence	Bacillus stratosphericus	2290	2290	100%	0.0	96.10%	1531	NR_042336.1
Bacillus aerius strain 24K 16S ribosomal RNA, partial sequence	Bacillus aerius	2287	2287	99%	0.0	96.28%	1466	NR_118439.1
Bacillus xiamenensis strain MCCC 1A00008 16S ribosomal RNA, partial sequence	Bacillus xiamenensis	2285	2285	100%	0.0	96.03%	1513	NR_148244.1
Bacillus stratosphericus strain 41KF2a 16S ribosomal RNA, partial sequence	Bacillus stratosphericus	2281	2281	99%	0.0	96.21%	1463	NR_118441.1
Bacillus safensis strain NBRC 100820 16S ribosomal RNA, partial sequence	Bacillus safensis	2279	2279	100%	0.0	95.96%	1474	NR_113945.1
Bacillus capparidis strain EGI 6500252 16S ribosomal RNA, partial sequence	Bacillus capparidis	2278	2278	100%	0.0	95.89%	1546	NR_156073.1
Bacillus pumilus strain NBRC 12092 16S ribosomal RNA, partial sequence	Bacillus pumilus	2274	2274	100%	0.0	95.88%	1474	NR_112637.1
Bacillus safensis FO-36b 16S ribosomal RNA, partial sequence	Bacillus safensis FO-36b	2272	2272	99%	0.0	96.20%	1434	NR_041794.1
Bacillus gobiensis strain FJAT-4402 16S ribosomal RNA, partial sequence	Bacillus gobiensis	2270	2270	98%	0.0	96.25%	1432	NR_147766.1
Bacillus zhangzhouensis strain MCCC 1A08372 16S ribosomal RNA, partial sequence	Bacillus zhangzhouensis	2268	2268	100%	0.0	95.81%	1513	NR_148786.1
Bacillus australimaris strain MCCC 1A05787 16S ribosomal RNA, partial sequence	Bacillus australimaris	2268	2268	100%	0.0	95.82%	1513	NR_148787.1

Description	Scientific Name	Max Score	Total Score	Query Cover	E value	Per. Ident	Acc. Len	Accession
Bacillus pumilus strain ATCC 7061 16S ribosomal RNA, partial sequence	Bacillus pumilus	2266	2266	99%	0.0	96.12%	1434	NR_043242.1
Bacillus haikouensis strain C-89 16S ribosomal RNA, partial sequence	Bacillus haikouensis	2265	2265	100%	0.0	95.74%	1473	NR_148273.1
Mesobacillus aurantius strain JC1013 16S ribosomal RNA, partial sequence	Mesobacillus aurantius	2257	2257	99%	0.0	95.90%	1444	NR_180197.1
Rossellomorea aquimaris strain TF-12 16S ribosomal RNA, partial sequence	Rossellomorea aquimaris	2250	2250	100%	0.0	95.54%	1507	NR_025241.1
Bacillus salacetis strain SKP7-4 16S ribosomal RNA, partial sequence	Bacillus salacetis	2242	2242	100%	0.0	95.47%	1431	NR_179253.1
Rossellomorea marisflavi strain TF-11 16S ribosomal RNA, partial sequence	Rossellomorea marisflavi	2237	2237	100%	0.0	95.39%	1506	NR_025240.1
Rossellomorea marisflavi strain TF-11 16S ribosomal RNA, partial sequence	Rossellomorea marisflavi	2235	2235	99%	0.0	95.57%	1466	NR_118437.1
Rossellomorea arthrocnemi strain EAR8 16S ribosomal RNA, partial sequence	Rossellomorea arthrocnemi	2233	2233	100%	0.0	95.32%	1474	NR_181775.1
Bacillus carboniphilus strain JCM9731 16S ribosomal RNA, partial sequence	Bacillus carboniphilus	2233	2233	100%	0.0	95.34%	1504	NR_024690.1
Bacillus paralicheniformis strain KJ-16 16S ribosomal RNA, partial sequence	Bacillus paralicheniformis	2228	2228	87%	0.0	99.59%	1327	NR_137421.1
Heyndrickxia acidicola strain 105-2 16S ribosomal RNA, partial sequence	Heyndrickxia acidicola	2224	2224	100%	0.0	95.19%	1548	NR_041942.1
Cytobacillus massiliigabonensis strain Marseille-P2639 16S ribosomal RNA, partial sequence	Cytobacillus massiliigabonensis	2215	2215	100%	0.0	95.11%	1514	NR_179554.1
Bacillus timonensis strain 10403023 16S ribosomal RNA, partial sequence	Bacillus timonensis	2207	2207	100%	0.0	94.98%	1472	NR_133024.1
Litchfieldia sinesaloumensis strain Marseille-P3516 16S ribosomal RNA, partial sequence	Bacillus sinesaloumensis	2206	2206	100%	0.0	94.91%	1474	NR_147383.1
Mangrovibacillus cuniculi strain R1DC41 16S ribosomal RNA, partial sequence	Mangrovibacillus cuniculi	2204	2204	100%	0.0	94.98%	1549	NR_181118.1
Falsibacillus albus strain GY 10110 16S	Falsibacillus albus	2202	2202	100%	0.0	94.90%	1533	NR_171509.1

Description	Scientific Name	Max Score	Total Score	Query Cover	E value	Per. Ident	Acc. Len	Accession
ribosomal RNA, partial sequence								
Heyndrickxia shackletonii strain LMG 18435 16S ribosomal RNA, partial sequence	Heyndrickxia shackletonii	2202	2202	100%	0.0	94.90%	1503	NR_025373.1
Fredinandcohnia onubensis strain 0911MAR22V3 16S ribosomal RNA, partial sequence	Fredinandcohnia onubensis	2191	2191	99%	0.0	95.00%	1429	NR_149252.1
Heyndrickxia camelliae strain 7578-1 16S ribosomal RNA, partial sequence	Heyndrickxia camelliae	2191	2191	100%	0.0	94.77%	1549	NR_159341.1
Metabacillus galliciensis strain BFLP-1 16S ribosomal RNA, partial sequence	Metabacillus galliciensis	2187	2187	100%	0.0	94.76%	1490	NR_116886.1
Bacillus isabeliae strain CVS-8 16S ribosomal RNA, partial sequence	Bacillus isabeliae	2183	2183	96%	0.0	95.86%	1523	NR_042619.1
Fredinandcohnia salidurans strain KNUC7312 16S ribosomal RNA, partial sequence	Fredinandcohnia salidurans	2183	2183	100%	0.0	94.69%	1481	NR_179035.1
Metabacillus herbersteinensis strain D-1,5 16S ribosomal RNA, partial sequence	Metabacillus herbersteinensis	2180	2180	100%	0.0	94.62%	1464	NR_042286.1
Fredinandcohnia humi strain LMG 22167 16S ribosomal RNA, partial sequence	Fredinandcohnia humi	2180	2180	100%	0.0	94.62%	1504	NR_025626.1
Cytobacillus firmus strain IAM 12464 16S ribosomal RNA, partial sequence	Cytobacillus firmus	2180	2180	100%	0.0	94.69%	1483	NR_025842.1
Bacillus salis strain ES3 16S ribosomal RNA, partial sequence	Bacillus salis	2178	2178	100%	0.0	94.62%	1515	NR_179406.1
Metabacillus idriensis strain SMC 4352-2 16S ribosomal RNA, partial sequence	Metabacillus idriensis	2178	2178	99%	0.0	94.86%	1437	NR_043268.1
Bacillus pumilus strain SBMP2 16S ribosomal RNA, partial sequence	Bacillus pumilus	2176	2176	98%	0.0	95.04%	1456	NR_118381.1
Bacillus mesophilum strain IITR-54 16S ribosomal RNA, partial sequence	Bacillus mesophilum	2176	2176	100%	0.0	94.63%	1448	NR_178489.1
Mesobacillus foraminis strain CV53 16S ribosomal RNA, partial sequence	Mesobacillus foraminis	2176	2176	100%	0.0	94.62%	1534	NR_042274.1
Cytobacillus firmus strain NBRC 15306 16S ribosomal RNA, partial sequence	Cytobacillus firmus	2174	2174	100%	0.0	94.48%	1477	NR_112635.1
Cytobacillus gottheilii strain WCC 4585 16S ribosomal	Cytobacillus gottheilii	2174	2174	100%	0.0	94.56%	1512	NR_108491.1

Description	Scientific Name	Max Score	Total Score	Query Cover	E value	Per. Ident	Acc. Len	Accession
RNA, partial sequence								
Cytobacillus praedii strain FJAT-25547 16S ribosomal RNA, partial sequence	Cytobacillus praedii	2174	2174	99%	0.0	94.86%	1420	NR_157745.1
Bacillus nitroreducens strain GSS08 16S ribosomal RNA, partial sequence	Bacillus nitroreducens	2174	2174	100%	0.0	94.60%	1453	NR_178876.1
Heyndrickxia sporothermodurans strain M215 16S ribosomal RNA, partial sequence	Heyndrickxia sporothermodurans	2174	2174	100%	0.0	94.56%	1497	NR_026010.1
Bacillus oleivorans strain JC228 16S ribosomal RNA, partial sequence	Bacillus oleivorans	2172	2172	99%	0.0	94.86%	1400	NR_134703.1
Rossellomorea vietnamensis strain 15-1 16S ribosomal RNA, partial sequence	Rossellomorea vietnamensis	2170	2170	98%	0.0	94.87%	1388	NR_024808.1

Graphic Summary

Distribution of the top 100 Blast Hits on 100 subject sequences

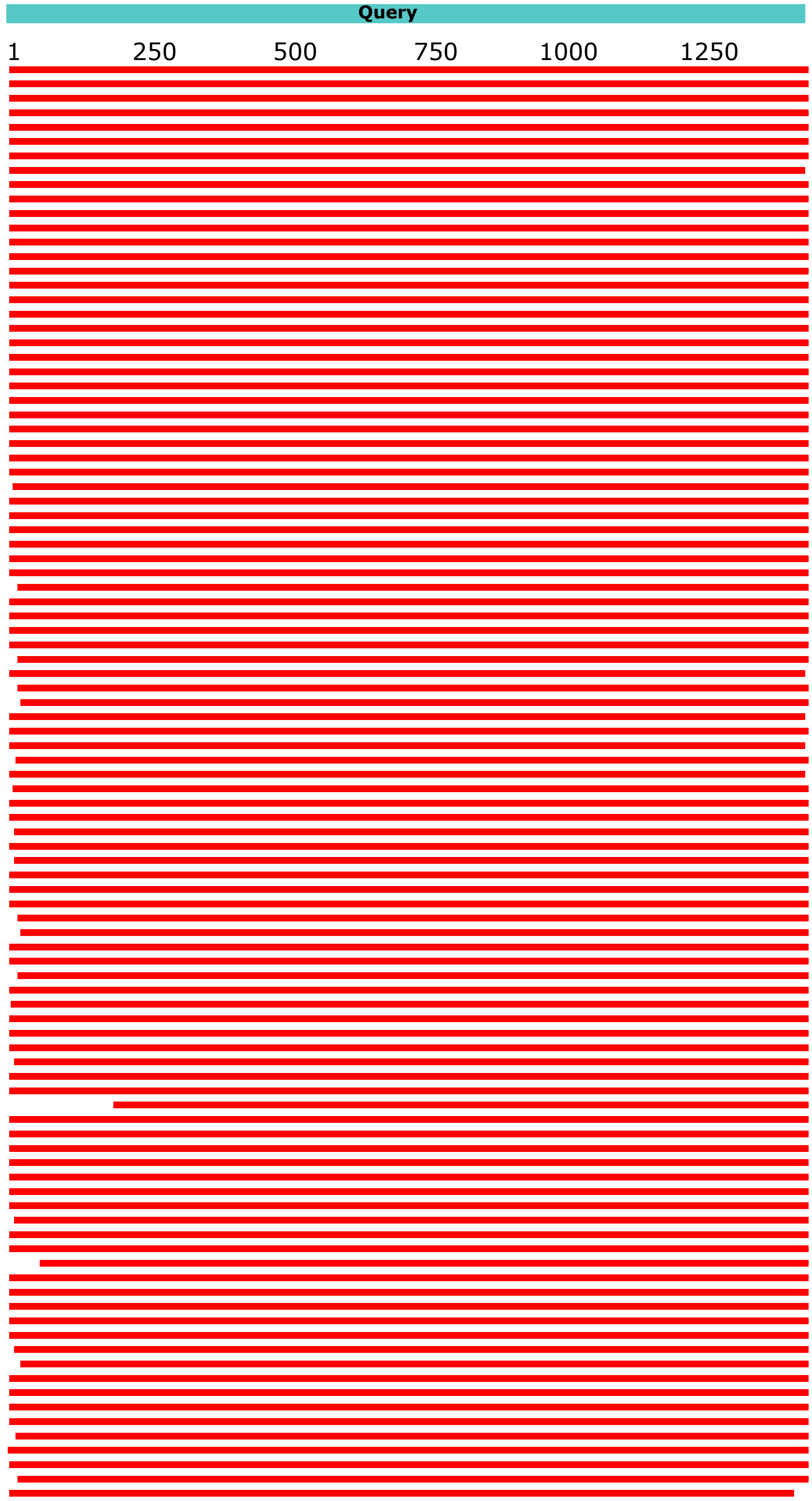

Alignments

Alignment view Pairwise ☐ CDS feature Restore defaults

Bacillus licheniformis strain BCRC 11702 16S ribosomal RNA, partial sequence

Sequence ID: **NR_116023.1** Length: 1468 Number of Matches: 1

Range 1: 10 to 1417

Score	Expect	Identities	Gaps	Strand	Frame
2553 bits(1382)	0.0()	1400/1408(99%)	4/1408(0%)	Plus/Plus	

```
Query  6    GCGG-GTG-CT-ATACATGC-AGTCGAGCGGACCGACGGGAGCTTGCTCCCTTAGGTCAG  61
            |||| ||| || |||||||| |||||||||||||||||||||||||||||||||||||||
Sbjct  10   GCGGCGTGCCTAATACATGCAAGTCGAGCGGACCGACGGGAGCTTGCTCCCTTAGGTCAG  69

Query  62   CGGCGGACGGGTGAGTAACACGTGGGTAACCTGCCTGTAAGACTGGGATAACTCCGGGAA  121
            ||||||||||||||||||||||||||||||||||||||||||||||||||||||||||||
Sbjct  70   CGGCGGACGGGTGAGTAACACGTGGGTAACCTGCCTGTAAGACTGGGATAACTCCGGGAA  129
```

```
Query  122   ACCGGGGCTAATACCGGATGCTTGATTGAACCGCATGGTTCAATCATAAAAGGTGGCTTT  181
             ||||||||||||||||||||||||||||||||||||||||||||||||||||||||||||
Sbjct  130   ACCGGGGCTAATACCGGATGCTTGATTGAACCGCATGGTTCAATCATAAAAGGTGGCTTT  189

Query  182   TAGCTACCACTTACAGATGGACCCGCGGCGCATTAGCTAGTTGGTGAGGTAACGGCTCAC  241
             ||||||||||||||||||||||||||||||||||||||||||||||||||||||||||||
Sbjct  190   TAGCTACCACTTACAGATGGACCCGCGGCGCATTAGCTAGTTGGTGAGGTAACGGCTCAC  249

Query  242   CAAGGCGACGATGCGTAGCCGACCTGAGAGGGTGATCGGCCACACTGGGACTGAGACACG  301
             ||||||||||||||||||||||||||||||||||||||||||||||||||||||||||||
Sbjct  250   CAAGGCGACGATGCGTAGCCGACCTGAGAGGGTGATCGGCCACACTGGGACTGAGACACG  309

Query  302   GCCCAGACTCCTACGGGAGGCAGCAGTAGGGAATCTTCCGCAATGGACGAAAGTCTGACG  361
             ||||||||||||||||||||||||||||||||||||||||||||||||||||||||||||
Sbjct  310   GCCCAGACTCCTACGGGAGGCAGCAGTAGGGAATCTTCCGCAATGGACGAAAGTCTGACG  369

Query  362   GAGCAACGCCGCGTGAGTGATGAAGGTTTTCGGGTCGTAAAACTCTGTTGTTAGGGAAGA  421
             ||||||||||||||||||||||||||||||||| ||||||||||||||||||||||||||
Sbjct  370   GAGCAACGCCGCGTGAGTGATGAAGGTTTTCGGATCGTAAAACTCTGTTGTTAGGGAAGA  429

Query  422   ACAAGTGCCGTTTGAATAGGGCGGCACCTTGACGGTACCTAACCAGAAAGCCACGGCTAA  481
             |||||| ||||| ||||||||||| |||||||||||||||||||||||||||||||||||
Sbjct  430   ACAAGTACCGTTCGAATAGGGCGGTACCTTGACGGTACCTAACCAGAAAGCCACGGCTAA  489

Query  482   CTACGTGCCAGCAGCCGCGGTAATACGTAGGTGGCAAGCGTTGTCCGGAATTATTGGGCG  541
             ||||||||||||||||||||||||||||||||||||||||||||||||||||||||||||
Sbjct  490   CTACGTGCCAGCAGCCGCGGTAATACGTAGGTGGCAAGCGTTGTCCGGAATTATTGGGCG  549

Query  542   TAAAGCGCGCGCAGGCGGTTTCTTAAGTCTGATGTGAAAGCCCCCGGCTCAACCGGGGAG  601
             ||||||||||||||||||||||||||||||||||||||||||||||||||||||||||||
Sbjct  550   TAAAGCGCGCGCAGGCGGTTTCTTAAGTCTGATGTGAAAGCCCCCGGCTCAACCGGGGAG  609

Query  602   GGTCATTGGAAACTGGGGAACTTGAGTGCAGAAGAGGAGAGTGGAATTCCACGTGTAGCG  661
             ||||||||||||||||||||||||||||||||||||||||||||||||||||||||||||
Sbjct  610   GGTCATTGGAAACTGGGGAACTTGAGTGCAGAAGAGGAGAGTGGAATTCCACGTGTAGCG  669

Query  662   GTGAAATGCGTAGAGATGTGGAGGAACACCAGTGGCGAAGGCGACTCTCTGGTCTGTAAC  721
             ||||||||||||||||||||||||||||||||||||||||||||||||||||||||||||
Sbjct  670   GTGAAATGCGTAGAGATGTGGAGGAACACCAGTGGCGAAGGCGACTCTCTGGTCTGTAAC  729

Query  722   TGACGCTGAGGCGCGAAAGCGTGGGGAGCGAACAGGATTAGATACCCTGGTAGTCCACGC  781
             ||||||||||||||||||||||||||||||||||||||||||||||||||||||||||||
Sbjct  730   TGACGCTGAGGCGCGAAAGCGTGGGGAGCGAACAGGATTAGATACCCTGGTAGTCCACGC  789

Query  782   CGTAAACGATGAGTGCTAAGTGTTAGAGGGTTTCCGCCCTTTAGTGCTGCAGCAAACGCA  841
             ||||||||||||||||||||||||||||||||||||||||||||||||||||||||||||
Sbjct  790   CGTAAACGATGAGTGCTAAGTGTTAGAGGGTTTCCGCCCTTTAGTGCTGCAGCAAACGCA  849

Query  842   TTAAGCACTCCGCCTGGGGAGTACGGTCGCAAGACTGAAACTCAAAGGAATTGACGGGGG  901
             ||||||||||||||||||||||||||||||||||||||||||||||||||||||||||||
Sbjct  850   TTAAGCACTCCGCCTGGGGAGTACGGTCGCAAGACTGAAACTCAAAGGAATTGACGGGGG  909

Query  902   CCCGCACAAGCGGTGGAGCATGTGGTTTAATTCGAAGCAACGCGAAGAACCTTACCAGGT  961
             ||||||||||||||||||||||||||||||||||||||||||||||||||||||||||||
Sbjct  910   CCCGCACAAGCGGTGGAGCATGTGGTTTAATTCGAAGCAACGCGAAGAACCTTACCAGGT  969

Query  962   CTTGACATCCTCTGACAACCCTAGAGATAGGGCTTCCCCTTCGGGGGCAGAGTGACAGGT  1021
             ||||||||||||||||||||||||||||||||||||||||||||||||||||||||||||
Sbjct  970   CTTGACATCCTCTGACAACCCTAGAGATAGGGCTTCCCCTTCGGGGGCAGAGTGACAGGT  1029

Query  1022  GGTGCATGGTTGTCGTCAGCTCGTGTCGTGAGATGTTGGGTTAAGTCCCGCAACGAGCGC  1081
             ||||||||||||||||||||||||||||||||||||||||||||||||||||||||||||
Sbjct  1030  GGTGCATGGTTGTCGTCAGCTCGTGTCGTGAGATGTTGGGTTAAGTCCCGCAACGAGCGC  1089

Query  1082  AACCCTTGATCTTAGTTGCCAGCATTCAGTTGGGCACTCTAAGGTGACTGCCGGTGACAA  1141
             ||||||||||||||||||||||||||||||||||||||||||||||||||||||||||||
Sbjct  1090  AACCCTTGATCTTAGTTGCCAGCATTCAGTTGGGCACTCTAAGGTGACTGCCGGTGACAA  1149

Query  1142  ACCGGAGGAAGGTGGGGATGACGTCAAATCATCATGCCCCTTATGACCTGGGCTACACAC  1201
             ||||||||||||||||||||||||||||||||||||||||||||||||||||||||||||
Sbjct  1150  ACCGGAGGAAGGTGGGGATGACGTCAAATCATCATGCCCCTTATGACCTGGGCTACACAC  1209

Query  1202  GTGCTACAATGGGCAGAACAAAGGGCAGCGAAGCCGCGAGGCTAAGCCAATCCCACAAAT  1261
             ||||||||||||||||||||||||||||||||||||||||||||||||||||||||||||
Sbjct  1210  GTGCTACAATGGGCAGAACAAAGGGCAGCGAAGCCGCGAGGCTAAGCCAATCCCACAAAT  1269

Query  1262  CTGTTCTCAGTTCGGATCGCAGTCTGCAACTCGACTGCGTGAAGCTGGAATCGCTAGTAA  1321
             ||||||||||||||||||||||||||||||||||||||||||||||||||||||||||||
Sbjct  1270  CTGTTCTCAGTTCGGATCGCAGTCTGCAACTCGACTGCGTGAAGCTGGAATCGCTAGTAA  1329

Query  1322  TCGCGGATCAGCATGCCGCGGTGAATACGTTCCCGGGCCTTGTACACACCGCCCGTCACA  1381
             ||||||||||||||||||||||||||||||||||||||||||||||||||||||||||||
Sbjct  1330  TCGCGGATCAGCATGCCGCGGTGAATACGTTCCCGGGCCTTGTACACACCGCCCGTCACA  1389

Query  1382  CCACGAGAGTTTGTAACACCCGAAGTCG  1409
             ||||||||||||||||||||||||||||
Sbjct  1390  CCACGAGAGTTTGTAACACCCGAAGTCG  1417
```

Bacillus licheniformis strain DSM 13 16S ribosomal RNA, partial sequence

Sequence ID: **NR_118996.1** Length: 1545 Number of Matches: 1

Range 1: 32 to 1439

Score	Expect	Identities	Gaps	Strand	Frame
2553 bits(1382)	0.0()	1400/1408(99%)	4/1408(0%)	Plus/Plus	

```
Query  6     GCGG-GTG-CT-ATACATGC-AGTCGAGCGGACCGACGGGAGCTTGCTCCCTTAGGTCAG  61
             |||| ||| || |||||||| |||||||||||||||||||||||||||||||||||||||
Sbjct  32    GCGGCGTGCCTAATACATGCAAGTCGAGCGGACCGACGGGAGCTTGCTCCCTTAGGTCAG  91
```

```
Query  62    CGGCGGACGGGTGAGTAACACGTGGGTAACCTGCCTGTAAGACTGGGATAACTCCGGGAA  121
             ||||||||||||||||||||||||||||||||||||||||||||||||||||||||||||
Sbjct  92    CGGCGGACGGGTGAGTAACACGTGGGTAACCTGCCTGTAAGACTGGGATAACTCCGGGAA  151

Query  122   ACCGGGGCTAATACCGGATGCTTGATTGAACCGCATGGTTCAATCATAAAAGGTGGCTTT  181
             ||||||||||||||||||||||||||||||||||||||||||||||||||||||||||||
Sbjct  152   ACCGGGGCTAATACCGGATGCTTGATTGAACCGCATGGTTCAATCATAAAAGGTGGCTTT  211

Query  182   TAGCTACCACTTACAGATGGACCCGCGGCGCATTAGCTAGTTGGTGAGGTAACGGCTCAC  241
             ||||||||||||||||||||||||||||||||||||||||||||||||||||||||||||
Sbjct  212   TAGCTACCACTTACAGATGGACCCGCGGCGCATTAGCTAGTTGGTGAGGTAACGGCTCAC  271

Query  242   CAAGGCGACGATGCGTAGCCGACCTGAGAGGGTGATCGGCCACACTGGGACTGAGACACG  301
             ||||||||||||||||||||||||||||||||||||||||||||||||||||||||||||
Sbjct  272   CAAGGCGACGATGCGTAGCCGACCTGAGAGGGTGATCGGCCACACTGGGACTGAGACACG  331

Query  302   GCCCAGACTCCTACGGGAGGCAGCAGTAGGGAATCTTCCGCAATGGACGAAAGTCTGACG  361
             ||||||||||||||||||||||||||||||||||||||||||||||||||||||||||||
Sbjct  332   GCCCAGACTCCTACGGGAGGCAGCAGTAGGGAATCTTCCGCAATGGACGAAAGTCTGACG  391

Query  362   GAGCAACGCCGCGTGAGTGATGAAGGTTTTCGGGTCGTAAAACTCTGTTGTTAGGGAAGA  421
             ||||||||||||||||||||||||||||||||| ||||||||||||||||||||||||||
Sbjct  392   GAGCAACGCCGCGTGAGTGATGAAGGTTTTCGGATCGTAAAACTCTGTTGTTAGGGAAGA  451

Query  422   ACAAGTGCCGTTTGAATAGGGCGGCACCTTGACGGTACCTAACCAGAAAGCCACGGCTAA  481
             |||||| ||||| ||||||||||| |||||||||||||||||||||||||||||||||||
Sbjct  452   ACAAGTACCGTTCGAATAGGGCGGTACCTTGACGGTACCTAACCAGAAAGCCACGGCTAA  511

Query  482   CTACGTGCCAGCAGCCGCGGTAATACGTAGGTGGCAAGCGTTGTCCGGAATTATTGGGCG  541
             ||||||||||||||||||||||||||||||||||||||||||||||||||||||||||||
Sbjct  512   CTACGTGCCAGCAGCCGCGGTAATACGTAGGTGGCAAGCGTTGTCCGGAATTATTGGGCG  571

Query  542   TAAAGCGCGCGCAGGCGGTTTCTTAAGTCTGATGTGAAAGCCCCCGGCTCAACCGGGGAG  601
             ||||||||||||||||||||||||||||||||||||||||||||||||||||||||||||
Sbjct  572   TAAAGCGCGCGCAGGCGGTTTCTTAAGTCTGATGTGAAAGCCCCCGGCTCAACCGGGGAG  631

Query  602   GGTCATTGGAAACTGGGGAACTTGAGTGCAGAAGAGGAGAGTGGAATTCCACGTGTAGCG  661
             ||||||||||||||||||||||||||||||||||||||||||||||||||||||||||||
Sbjct  632   GGTCATTGGAAACTGGGGAACTTGAGTGCAGAAGAGGAGAGTGGAATTCCACGTGTAGCG  691

Query  662   GTGAAATGCGTAGAGATGTGGAGGAACACCAGTGGCGAAGGCGACTCTCTGGTCTGTAAC  721
             ||||||||||||||||||||||||||||||||||||||||||||||||||||||||||||
Sbjct  692   GTGAAATGCGTAGAGATGTGGAGGAACACCAGTGGCGAAGGCGACTCTCTGGTCTGTAAC  751

Query  722   TGACGCTGAGGCGCGAAAGCGTGGGGAGCGAACAGGATTAGATACCCTGGTAGTCCACGC  781
             ||||||||||||||||||||||||||||||||||||||||||||||||||||||||||||
Sbjct  752   TGACGCTGAGGCGCGAAAGCGTGGGGAGCGAACAGGATTAGATACCCTGGTAGTCCACGC  811

Query  782   CGTAAACGATGAGTGCTAAGTGTTAGAGGGTTTCCGCCCTTTAGTGCTGCAGCAAACGCA  841
             ||||||||||||||||||||||||||||||||||||||||||||||||||||||||||||
Sbjct  812   CGTAAACGATGAGTGCTAAGTGTTAGAGGGTTTCCGCCCTTTAGTGCTGCAGCAAACGCA  871

Query  842   TTAAGCACTCCGCCTGGGGAGTACGGTCGCAAGACTGAAACTCAAAGGAATTGACGGGGG  901
             ||||||||||||||||||||||||||||||||||||||||||||||||||||||||||||
Sbjct  872   TTAAGCACTCCGCCTGGGGAGTACGGTCGCAAGACTGAAACTCAAAGGAATTGACGGGGG  931

Query  902   CCCGCACAAGCGGTGGAGCATGTGGTTTAATTCGAAGCAACGCGAAGAACCTTACCAGGT  961
             ||||||||||||||||||||||||||||||||||||||||||||||||||||||||||||
Sbjct  932   CCCGCACAAGCGGTGGAGCATGTGGTTTAATTCGAAGCAACGCGAAGAACCTTACCAGGT  991

Query  962   CTTGACATCCTCTGACAACCCTAGAGATAGGGCTTCCCCTTCGGGGGCAGAGTGACAGGT  1021
             ||||||||||||||||||||||||||||||||||||||||||||||||||||||||||||
Sbjct  992   CTTGACATCCTCTGACAACCCTAGAGATAGGGCTTCCCCTTCGGGGGCAGAGTGACAGGT  1051

Query  1022  GGTGCATGGTTGTCGTCAGCTCGTGTCGTGAGATGTTGGGTTAAGTCCCGCAACGAGCGC  1081
             ||||||||||||||||||||||||||||||||||||||||||||||||||||||||||||
Sbjct  1052  GGTGCATGGTTGTCGTCAGCTCGTGTCGTGAGATGTTGGGTTAAGTCCCGCAACGAGCGC  1111

Query  1082  AACCCTTGATCTTAGTTGCCAGCATTCAGTTGGGCACTCTAAGGTGACTGCCGGTGACAA  1141
             ||||||||||||||||||||||||||||||||||||||||||||||||||||||||||||
Sbjct  1112  AACCCTTGATCTTAGTTGCCAGCATTCAGTTGGGCACTCTAAGGTGACTGCCGGTGACAA  1171

Query  1142  ACCGGAGGAAGGTGGGGATGACGTCAAATCATCATGCCCCTTATGACCTGGGCTACACAC  1201
             ||||||||||||||||||||||||||||||||||||||||||||||||||||||||||||
Sbjct  1172  ACCGGAGGAAGGTGGGGATGACGTCAAATCATCATGCCCCTTATGACCTGGGCTACACAC  1231

Query  1202  GTGCTACAATGGGCAGAACAAAGGGCAGCGAAGCCGCGAGGCTAAGCCAATCCCACAAAT  1261
             ||||||||||||||||||||||||||||||||||||||||||||||||||||||||||||
Sbjct  1232  GTGCTACAATGGGCAGAACAAAGGGCAGCGAAGCCGCGAGGCTAAGCCAATCCCACAAAT  1291

Query  1262  CTGTTCTCAGTTCGGATCGCAGTCTGCAACTCGACTGCGTGAAGCTGGAATCGCTAGTAA  1321
             ||||||||||||||||||||||||||||||||||||||||||||||||||||||||||||
Sbjct  1292  CTGTTCTCAGTTCGGATCGCAGTCTGCAACTCGACTGCGTGAAGCTGGAATCGCTAGTAA  1351

Query  1322  TCGCGGATCAGCATGCCGCGGTGAATACGTTCCCGGGCCTTGTACACACCGCCCGTCACA  1381
             ||||||||||||||||||||||||||||||||||||||||||||||||||||||||||||
Sbjct  1352  TCGCGGATCAGCATGCCGCGGTGAATACGTTCCCGGGCCTTGTACACACCGCCCGTCACA  1411

Query  1382  CCACGAGAGTTTGTAACACCCGAAGTCG  1409
             ||||||||||||||||||||||||||||
Sbjct  1412  CCACGAGAGTTTGTAACACCCGAAGTCG  1439
```

Bacillus licheniformis strain NBRC 12200 16S ribosomal RNA, partial sequence

Sequence ID: **NR_113588.1** Length: 1475 Number of Matches: 1

Range 1: 12 to 1419

Score	Expect	Identities	Gaps	Strand	Frame
2547 bits(1379)	0.0()	1398/1408(99%)	4/1408(0%)	Plus/Plus	

```
Query  6     GCGG-GTG-CT-ATACATGC-AGTCGAGCGGACCGACGGGAGCTTGCTCCCTTAGGTCAG  61
             |||| ||| || |||||||| |||||||||||||||||||||||||||||||||||||||
Sbjct  12    GCGGCGTGCCTAATACATGCAAGTCGAGCGGACCGACGGGAGCTTGCTCCCTTAGGTCAG  71

Query  62    CGGCGGACGGGTGAGTAACACGTGGGTAACCTGCCTGTAAGACTGGGATAACTCCGGGAA  121
             ||||||||||||||||||||||||||||||||||||||||||||||||||||||||||||
Sbjct  72    CGGCGGACGGGTGAGTAACACGTGGGTAACCTGCCTGTAAGACTGGGATAACTCCGGGAA  131

Query  122   ACCGGGGCTAATACCGGATGCTTGATTGAACCGCATGGTTCAATCATAAAAGGTGGCTTT  181
             ||||||||||||||||||||||||||||||||||||||||||||||||||||||||||||
Sbjct  132   ACCGGGGCTAATACCGGATGCTTGATTGAACCGCATGGTTCAATCATAAAAGGTGGCTTT  191

Query  182   TAGCTACCACTTACAGATGGACCCGCGGCGCATTAGCTAGTTGGTGAGGTAACGGCTCAC  241
              |||||||||||||||||||||||||||||||||||||||||||||||||||||||||||
Sbjct  192   NAGCTACCACTTACAGATGGACCCGCGGCGCATTAGCTAGTTGGTGAGGTAACGGCTCAC  251

Query  242   CAAGGCGACGATGCGTAGCCGACCTGAGAGGGTGATCGGCCACACTGGGACTGAGACACG  301
             ||||||||||||||||||||||||||||||||||||||||||||||||||||||||||||
Sbjct  252   CAAGGCGACGATGCGTAGCCGACCTGAGAGGGTGATCGGCCACACTGGGACTGAGACACG  311

Query  302   GCCCAGACTCCTACGGGAGGCAGCAGTAGGGAATCTTCCGCAATGGACGAAAGTCTGACG  361
             ||||||||||||||||||||||||||||||||||||||||||||||||||||||||||||
Sbjct  312   GCCCAGACTCCTACGGGAGGCAGCAGTAGGGAATCTTCCGCAATGGACGAAAGTCTGACG  371

Query  362   GAGCAACGCCGCGTGAGTGATGAAGGTTTTCGGGTCGTAAAACTCTGTTGTTAGGGAAGA  421
             ||||||||||||||||||||||||||||||||| ||||||||||||||||||||||||||
Sbjct  372   GAGCAACGCCGCGTGAGTGATGAAGGTTTTCGGATCGTAAAACTCTGTTGTTAGGGAAGA  431

Query  422   ACAAGTGCCGTTTGAATAGGGCGGCACCTTGACGGTACCTAACCAGAAAGCCACGGCTAA  481
             |||||| ||||| ||||||||||| |||||||||||||||||||||||||||||||||||
Sbjct  432   ACAAGTACCGTTCGAATAGGGCGGNACCTTGACGGTACCTAACCAGAAAGCCACGGCTAA  491

Query  482   CTACGTGCCAGCAGCCGCGGTAATACGTAGGTGGCAAGCGTTGTCCGGAATTATTGGGCG  541
             ||||||||||||||||||||||||||||||||||||||||||||||||||||||||||||
Sbjct  492   CTACGTGCCAGCAGCCGCGGTAATACGTAGGTGGCAAGCGTTGTCCGGAATTATTGGGCG  551

Query  542   TAAAGCGCGCGCAGGCGGTTTCTTAAGTCTGATGTGAAAGCCCCCGGCTCAACCGGGGAG  601
             ||||||||||||||||||||||||||||||||||||||||||||||||||||||||||||
Sbjct  552   TAAAGCGCGCGCAGGCGGTTTCTTAAGTCTGATGTGAAAGCCCCCGGCTCAACCGGGGAG  611

Query  602   GGTCATTGGAAACTGGGGAACTTGAGTGCAGAAGAGGAGAGTGGAATTCCACGTGTAGCG  661
             ||||||||||||||||||||||||||||||||||||||||||||||||||||||||||||
Sbjct  612   GGTCATTGGAAACTGGGGAACTTGAGTGCAGAAGAGGAGAGTGGAATTCCACGTGTAGCG  671

Query  662   GTGAAATGCGTAGAGATGTGGAGGAACACCAGTGGCGAAGGCGACTCTCTGGTCTGTAAC  721
             ||||||||||||||||||||||||||||||||||||||||||||||||||||||||||||
Sbjct  672   GTGAAATGCGTAGAGATGTGGAGGAACACCAGTGGCGAAGGCGACTCTCTGGTCTGTAAC  731

Query  722   TGACGCTGAGGCGCGAAAGCGTGGGGAGCGAACAGGATTAGATACCCTGGTAGTCCACGC  781
             ||||||||||||||||||||||||||||||||||||||||||||||||||||||||||||
Sbjct  732   TGACGCTGAGGCGCGAAAGCGTGGGGAGCGAACAGGATTAGATACCCTGGTAGTCCACGC  791

Query  782   CGTAAACGATGAGTGCTAAGTGTTAGAGGGTTTCCGCCCTTTAGTGCTGCAGCAAACGCA  841
             ||||||||||||||||||||||||||||||||||||||||||||||||||||||||||||
Sbjct  792   CGTAAACGATGAGTGCTAAGTGTTAGAGGGTTTCCGCCCTTTAGTGCTGCAGCAAACGCA  851

Query  842   TTAAGCACTCCGCCTGGGGAGTACGGTCGCAAGACTGAAACTCAAAGGAATTGACGGGGG  901
             ||||||||||||||||||||||||||||||||||||||||||||||||||||||||||||
Sbjct  852   TTAAGCACTCCGCCTGGGGAGTACGGTCGCAAGACTGAAACTCAAAGGAATTGACGGGGG  911

Query  902   CCCGCACAAGCGGTGGAGCATGTGGTTTAATTCGAAGCAACGCGAAGAACCTTACCAGGT  961
             ||||||||||||||||||||||||||||||||||||||||||||||||||||||||||||
Sbjct  912   CCCGCACAAGCGGTGGAGCATGTGGTTTAATTCGAAGCAACGCGAAGAACCTTACCAGGT  971

Query  962   CTTGACATCCTCTGACAACCCTAGAGATAGGGCTTCCCCTTCGGGGGCAGAGTGACAGGT  1021
             |||||||||||||| |||||||||||||||||||||||||||||||||||||||||||||
Sbjct  972   CTTGACATCCTCTGNCAACCCTAGAGATAGGGCTTCCCCTTCGGGGGCAGAGTGACAGGT  1031

Query  1022  GGTGCATGGTTGTCGTCAGCTCGTGTCGTGAGATGTTGGGTTAAGTCCCGCAACGAGCGC  1081
             ||||||||||||||||||||||||||||||||||||||||||||||||||||||||||||
Sbjct  1032  GGTGCATGGTTGTCGTCAGCTCGTGTCGTGAGATGTTGGGTTAAGTCCCGCAACGAGCGC  1091

Query  1082  AACCCTTGATCTTAGTTGCCAGCATTCAGTTGGGCACTCTAAGGTGACTGCCGGTGACAA  1141
             ||||||||||||||||||||||||||||||||||||||||||||||||||||||||||||
Sbjct  1092  AACCCTTGATCTTAGTTGCCAGCATTCAGTTGGGCACTCTAAGGTGACTGCCGGTGACAA  1151

Query  1142  ACCGGAGGAAGGTGGGGATGACGTCAAATCATCATGCCCCTTATGACCTGGGCTACACAC  1201
             ||||||||||||||||||||||||||||||||||||||||||||||||||||||||||||
Sbjct  1152  ACCGGAGGAAGGTGGGGATGACGTCAAATCATCATGCCCCTTATGACCTGGGCTACACAC  1211

Query  1202  GTGCTACAATGGGCAGAACAAAGGGCAGCGAAGCCGCGAGGCTAAGCCAATCCCACAAAT  1261
             ||||||||||||||||||||||||||||||||||||||||||||||||||||||||||||
Sbjct  1212  GTGCTACAATGGGCAGAACAAAGGGCAGCGAAGCCGCGAGGCTAAGCCAATCCCACAAAT  1271

Query  1262  CTGTTCTCAGTTCGGATCGCAGTCTGCAACTCGACTGCGTGAAGCTGGAATCGCTAGTAA  1321
             ||||||||||||||||||||||||||||||||||||||||||||||||||||||||||||
Sbjct  1272  CTGTTCTCAGTTCGGATCGCAGTCTGCAACTCGACTGCGTGAAGCTGGAATCGCTAGTAA  1331

Query  1322  TCGCGGATCAGCATGCCGCGGTGAATACGTTCCCGGGCCTTGTACACACCGCCCGTCACA  1381
             ||||||||||||||||||||||||||||||||||||||||||||||||||||||||||||
Sbjct  1332  TCGCGGATCAGCATGCCGCGGTGAATACGTTCCCGGGCCTTGTACACACCGCCCGTCACA  1391

Query  1382  CCACGAGAGTTTGTAACACCCGAAGTCG  1409
             ||||||||||||||||||||||||||||
Sbjct  1392  CCACGAGAGTTTGTAACACCCGAAGTCG  1419
```

Bacillus licheniformis strain ATCC 14580 16S ribosomal RNA, partial sequence

Sequence ID: **NR_074923.1** Length: 1545 Number of Matches: 1

Range 1: 32 to 1439

Score	Expect	Identities	Gaps	Strand	Frame
2542 bits(1376)	0.0()	1398/1408(99%)	4/1408(0%)	Plus/Plus	

```
Query  6     GCGG-GTG-CT-ATACATGC-AGTCGAGCGGACCGACGGGAGCTTGCTCCCTTAGGTCAG  61
             |||| ||| || |||||||| |||||||||||||||||||||||||||||||||||||||
Sbjct  32    GCGGCGTGCCTAATACATGCAAGTCGAGCGGACCGACGGGAGCTTGCTCCCTTAGGTCAG  91

Query  62    CGGCGGACGGGTGAGTAACACGTGGGTAACCTGCCTGTAAGACTGGGATAACTCCGGGAA  121
             ||||||||||||||||||||||||||||||||||||||||||||||||||||||||||||
Sbjct  92    CGGCGGACGGGTGAGTAACACGTGGGTAACCTGCCTGTAAGACTGGGATAACTCCGGGAA  151

Query  122   ACCGGGGCTAATACCGGATGCTTGATTGAACCGCATGGTTCAATCATAAAAGGTGGCTTT  181
             ||||||||||||||||||||||||||||||||||||||||||||||||||||||||||||
Sbjct  152   ACCGGGGCTAATACCGGATGCTTGATTGAACCGCATGGTTCAATCATAAAAGGTGGCTTT  211

Query  182   TAGCTACCACTTACAGATGGACCCGCGGCGCATTAGCTAGTTGGTGAGGTAACGGCTCAC  241
             |||||||||||| |||||||||||||||||||||||||||||||||||||||||||||||
Sbjct  212   TAGCTACCACTTGCAGATGGACCCGCGGCGCATTAGCTAGTTGGTGAGGTAACGGCTCAC  271

Query  242   CAAGGCGACGATGCGTAGCCGACCTGAGAGGGTGATCGGCCACACTGGGACTGAGACACG  301
             ||||||||||||||||||||||||||||||||||||||||||||||||||||||||||||
Sbjct  272   CAAGGCGACGATGCGTAGCCGACCTGAGAGGGTGATCGGCCACACTGGGACTGAGACACG  331

Query  302   GCCCAGACTCCTACGGGAGGCAGCAGTAGGGAATCTTCCGCAATGGACGAAAGTCTGACG  361
             ||||||||||||||||||||||||||||||||||||||||||||||||||||||||||||
Sbjct  332   GCCCAGACTCCTACGGGAGGCAGCAGTAGGGAATCTTCCGCAATGGACGAAAGTCTGACG  391

Query  362   GAGCAACGCCGCGTGAGTGATGAAGGTTTTCGGGTCGTAAAACTCTGTTGTTAGGGAAGA  421
             ||||||||||||||||||||||||||||||||| ||||||||||||||||||||||||||
Sbjct  392   GAGCAACGCCGCGTGAGTGATGAAGGTTTTCGGATCGTAAAACTCTGTTGTTAGGGAAGA  451

Query  422   ACAAGTGCCGTTTGAATAGGGCGGCACCTTGACGGTACCTAACCAGAAAGCCACGGCTAA  481
             |||||| ||||| ||||||||||| |||||||||||||||||||||||||||||||||||
Sbjct  452   ACAAGTACCGTTCGAATAGGGCGGTACCTTGACGGTACCTAACCAGAAAGCCACGGCTAA  511

Query  482   CTACGTGCCAGCAGCCGCGGTAATACGTAGGTGGCAAGCGTTGTCCGGAATTATTGGGCG  541
             ||||||||||||||||||||||||||||||||||||||||||||||||||||||||||||
Sbjct  512   CTACGTGCCAGCAGCCGCGGTAATACGTAGGTGGCAAGCGTTGTCCGGAATTATTGGGCG  571

Query  542   TAAAGCGCGCGCAGGCGGTTTCTTAAGTCTGATGTGAAAGCCCCCGGCTCAACCGGGGAG  601
             ||||||||||||||||||||||||||||||||||||||||||||||||||||||||||||
Sbjct  572   TAAAGCGCGCGCAGGCGGTTTCTTAAGTCTGATGTGAAAGCCCCCGGCTCAACCGGGGAG  631

Query  602   GGTCATTGGAAACTGGGGAACTTGAGTGCAGAAGAGGAGAGTGGAATTCCACGTGTAGCG  661
             ||||||||||||||||||||||||||||||||||||||||||||||||||||||||||||
Sbjct  632   GGTCATTGGAAACTGGGGAACTTGAGTGCAGAAGAGGAGAGTGGAATTCCACGTGTAGCG  691

Query  662   GTGAAATGCGTAGAGATGTGGAGGAACACCAGTGGCGAAGGCGACTCTCTGGTCTGTAAC  721
             ||||||||||||||||||||||||||||||||||||||||||||||||||||||||||||
Sbjct  692   GTGAAATGCGTAGAGATGTGGAGGAACACCAGTGGCGAAGGCGACTCTCTGGTCTGTAAC  751

Query  722   TGACGCTGAGGCGCGAAAGCGTGGGGAGCGAACAGGATTAGATACCCTGGTAGTCCACGC  781
             ||||||||||||||||||||||||||||||||||||||||||||||||||||||||||||
Sbjct  752   TGACGCTGAGGCGCGAAAGCGTGGGGAGCGAACAGGATTAGATACCCTGGTAGTCCACGC  811

Query  782   CGTAAACGATGAGTGCTAAGTGTTAGAGGGTTTCCGCCCTTTAGTGCTGCAGCAAACGCA  841
             ||||||||||||||||||||||||||||||||||||||||||||||||||||||||||||
Sbjct  812   CGTAAACGATGAGTGCTAAGTGTTAGAGGGTTTCCGCCCTTTAGTGCTGCAGCAAACGCA  871

Query  842   TTAAGCACTCCGCCTGGGGAGTACGGTCGCAAGACTGAAACTCAAAGGAATTGACGGGGG  901
             ||||||||||||||||||||||||||||||||||||||||||||||||||||||||||||
Sbjct  872   TTAAGCACTCCGCCTGGGGAGTACGGTCGCAAGACTGAAACTCAAAGGAATTGACGGGGG  931

Query  902   CCCGCACAAGCGGTGGAGCATGTGGTTTAATTCGAAGCAACGCGAAGAACCTTACCAGGT  961
             ||||||||||||||||||||||||||||||||||||||||||||||||||||||||||||
Sbjct  932   CCCGCACAAGCGGTGGAGCATGTGGTTTAATTCGAAGCAACGCGAAGAACCTTACCAGGT  991

Query  962   CTTGACATCCTCTGACAACCCTAGAGATAGGGCTTCCCCTTCGGGGGCAGAGTGACAGGT  1021
             |||||||||||||| |||||||||||||||||||||||||||||||||||||||||||||
Sbjct  992   CTTGACATCCTCTGGCAACCCTAGAGATAGGGCTTCCCCTTCGGGGGCAGAGTGACAGGT  1051

Query  1022  GGTGCATGGTTGTCGTCAGCTCGTGTCGTGAGATGTTGGGTTAAGTCCCGCAACGAGCGC  1081
             ||||||||||||||||||||||||||||||||||||||||||||||||||||||||||||
Sbjct  1052  GGTGCATGGTTGTCGTCAGCTCGTGTCGTGAGATGTTGGGTTAAGTCCCGCAACGAGCGC  1111

Query  1082  AACCCTTGATCTTAGTTGCCAGCATTCAGTTGGGCACTCTAAGGTGACTGCCGGTGACAA  1141
             ||||||||||||||||||||||||||||||||||||||||||||||||||||||||||||
Sbjct  1112  AACCCTTGATCTTAGTTGCCAGCATTCAGTTGGGCACTCTAAGGTGACTGCCGGTGACAA  1171

Query  1142  ACCGGAGGAAGGTGGGGATGACGTCAAATCATCATGCCCCTTATGACCTGGGCTACACAC  1201
             ||||||||||||||||||||||||||||||||||||||||||||||||||||||||||||
Sbjct  1172  ACCGGAGGAAGGTGGGGATGACGTCAAATCATCATGCCCCTTATGACCTGGGCTACACAC  1231

Query  1202  GTGCTACAATGGGCAGAACAAAGGGCAGCGAAGCCGCGAGGCTAAGCCAATCCCACAAAT  1261
             ||||||||||||||||||||||||||||||||||||||||||||||||||||||||||||
Sbjct  1232  GTGCTACAATGGGCAGAACAAAGGGCAGCGAAGCCGCGAGGCTAAGCCAATCCCACAAAT  1291

Query  1262  CTGTTCTCAGTTCGGATCGCAGTCTGCAACTCGACTGCGTGAAGCTGGAATCGCTAGTAA  1321
             ||||||||||||||||||||||||||||||||||||||||||||||||||||||||||||
Sbjct  1292  CTGTTCTCAGTTCGGATCGCAGTCTGCAACTCGACTGCGTGAAGCTGGAATCGCTAGTAA  1351

Query  1322  TCGCGGATCAGCATGCCGCGGTGAATACGTTCCCGGGCCTTGTACACACCGCCCGTCACA  1381
             ||||||||||||||||||||||||||||||||||||||||||||||||||||||||||||
Sbjct  1352  TCGCGGATCAGCATGCCGCGGTGAATACGTTCCCGGGCCTTGTACACACCGCCCGTCACA  1411

Query  1382  CCACGAGAGTTTGTAACACCCGAAGTCG  1409
             ||||||||||||||||||||||||||||
Sbjct  1412  CCACGAGAGTTTGTAACACCCGAAGTCG  1439
```

Bacillus haynesii strain NRRL B-41327 16S ribosomal RNA, partial sequence
Sequence ID: **NR_157609.1** Length: 1508 Number of Matches: 1
Range 1: 32 to 1439

**Score**	**Expect**	**Identities**	**Gaps**	**Strand**	**Frame**
2536 bits(1373)	0.0()	1397/1408(99%)	4/1408(0%)	Plus/Plus	

```
Query  6     GCGG-GTG-CT-ATACATGC-AGTCGAGCGGACCGACGGGAGCTTGCTCCCTTAGGTCAG  61
             |||| ||| || |||||||| |||||||||||||||||||||||||||||||||||| ||
Sbjct  32    GCGGCGTGCCTAATACATGCAAGTCGAGCGGACCGACGGGAGCTTGCTCCCTTAGGTTAG  91

Query  62    CGGCGGACGGGTGAGTAACACGTGGGTAACCTGCCTGTAAGACTGGGATAACTCCGGGAA  121
             ||||||||||||||||||||||||||||||||||||||||||||||||||||||||||||
Sbjct  92    CGGCGGACGGGTGAGTAACACGTGGGTAACCTGCCTGTAAGACTGGGATAACTCCGGGAA  151

Query  122   ACCGGGGCTAATACCGGATGCTTGATTGAACCGCATGGTTCAATCATAAAAGGTGGCTTT  181
             |||||||||||||||||||||||||||||||||||||||||||| |||||||||||||||
Sbjct  152   ACCGGGGCTAATACCGGATGCTTGATTGAACCGCATGGTTCAATTATAAAAGGTGGCTTT  211

Query  182   TAGCTACCACTTACAGATGGACCCGCGGCGCATTAGCTAGTTGGTGAGGTAACGGCTCAC  241
             ||||||||||||||||||||||||||||||||||||||||||||||||||||||||||||
Sbjct  212   TAGCTACCACTTACAGATGGACCCGCGGCGCATTAGCTAGTTGGTGAGGTAACGGCTCAC  271

Query  242   CAAGGCGACGATGCGTAGCCGACCTGAGAGGGTGATCGGCCACACTGGGACTGAGACACG  301
             |||||| |||||||||||||||||||||||||||||||||||||||||||||||||||||
Sbjct  272   CAAGGCAACGATGCGTAGCCGACCTGAGAGGGTGATCGGCCACACTGGGACTGAGACACG  331

Query  302   GCCCAGACTCCTACGGGAGGCAGCAGTAGGGAATCTTCCGCAATGGACGAAAGTCTGACG  361
             ||||||||||||||||||||||||||||||||||||||||||||||||||||||||||||
Sbjct  332   GCCCAGACTCCTACGGGAGGCAGCAGTAGGGAATCTTCCGCAATGGACGAAAGTCTGACG  391

Query  362   GAGCAACGCCGCGTGAGTGATGAAGGTTTTCGGGTCGTAAAACTCTGTTGTTAGGGAAGA  421
             ||||||||||||||||||||||||||||||||| ||||||||||||||||||||||||||
Sbjct  392   GAGCAACGCCGCGTGAGTGATGAAGGTTTTCGGATCGTAAAACTCTGTTGTTAGGGAAGA  451

Query  422   ACAAGTGCCGTTTGAATAGGGCGGCACCTTGACGGTACCTAACCAGAAAGCCACGGCTAA  481
             |||||| ||||| ||||||||||| |||||||||||||||||||||||||||||||||||
Sbjct  452   ACAAGTACCGTTCGAATAGGGCGGTACCTTGACGGTACCTAACCAGAAAGCCACGGCTAA  511

Query  482   CTACGTGCCAGCAGCCGCGGTAATACGTAGGTGGCAAGCGTTGTCCGGAATTATTGGGCG  541
             ||||||||||||||||||||||||||||||||||||||||||||||||||||||||||||
Sbjct  512   CTACGTGCCAGCAGCCGCGGTAATACGTAGGTGGCAAGCGTTGTCCGGAATTATTGGGCG  571

Query  542   TAAAGCGCGCGCAGGCGGTTTCTTAAGTCTGATGTGAAAGCCCCCGGCTCAACCGGGGAG  601
             ||||||||||||||||||||||||||||||||||||||||||||||||||||||||||||
Sbjct  572   TAAAGCGCGCGCAGGCGGTTTCTTAAGTCTGATGTGAAAGCCCCCGGCTCAACCGGGGAG  631

Query  602   GGTCATTGGAAACTGGGGAACTTGAGTGCAGAAGAGGAGAGTGGAATTCCACGTGTAGCG  661
             ||||||||||||||||||||||||||||||||||||||||||||||||||||||||||||
Sbjct  632   GGTCATTGGAAACTGGGGAACTTGAGTGCAGAAGAGGAGAGTGGAATTCCACGTGTAGCG  691

Query  662   GTGAAATGCGTAGAGATGTGGAGGAACACCAGTGGCGAAGGCGACTCTCTGGTCTGTAAC  721
             ||||||||||||||||||||||||||||||||||||||||||||||||||||||||||||
Sbjct  692   GTGAAATGCGTAGAGATGTGGAGGAACACCAGTGGCGAAGGCGACTCTCTGGTCTGTAAC  751

Query  722   TGACGCTGAGGCGCGAAAGCGTGGGGAGCGAACAGGATTAGATACCCTGGTAGTCCACGC  781
             ||||||||||||||||||||||||||||||||||||||||||||||||||||||||||||
Sbjct  752   TGACGCTGAGGCGCGAAAGCGTGGGGAGCGAACAGGATTAGATACCCTGGTAGTCCACGC  811

Query  782   CGTAAACGATGAGTGCTAAGTGTTAGAGGGTTTCCGCCCTTTAGTGCTGCAGCAAACGCA  841
             ||||||||||||||||||||||||||||||||||||||||||||||||||||||||||||
Sbjct  812   CGTAAACGATGAGTGCTAAGTGTTAGAGGGTTTCCGCCCTTTAGTGCTGCAGCAAACGCA  871

Query  842   TTAAGCACTCCGCCTGGGGAGTACGGTCGCAAGACTGAAACTCAAAGGAATTGACGGGGG  901
             ||||||||||||||||||||||||||||||||||||||||||||||||||||||||||||
Sbjct  872   TTAAGCACTCCGCCTGGGGAGTACGGTCGCAAGACTGAAACTCAAAGGAATTGACGGGGG  931

Query  902   CCCGCACAAGCGGTGGAGCATGTGGTTTAATTCGAAGCAACGCGAAGAACCTTACCAGGT  961
             ||||||||||||||||||||||||||||||||||||||||||||||||||||||||||||
Sbjct  932   CCCGCACAAGCGGTGGAGCATGTGGTTTAATTCGAAGCAACGCGAAGAACCTTACCAGGT  991

Query  962   CTTGACATCCTCTGACAACCCTAGAGATAGGGCTTCCCCTTCGGGGGCAGAGTGACAGGT  1021
             ||||||||||||||||||||||||||||||||||||||||||||||||||||||||||||
Sbjct  992   CTTGACATCCTCTGACAACCCTAGAGATAGGGCTTCCCCTTCGGGGGCAGAGTGACAGGT  1051

Query  1022  GGTGCATGGTTGTCGTCAGCTCGTGTCGTGAGATGTTGGGTTAAGTCCCGCAACGAGCGC  1081
             ||||||||||||||||||||||||||||||||||||||||||||||||||||||||||||
Sbjct  1052  GGTGCATGGTTGTCGTCAGCTCGTGTCGTGAGATGTTGGGTTAAGTCCCGCAACGAGCGC  1111

Query  1082  AACCCTTGATCTTAGTTGCCAGCATTCAGTTGGGCACTCTAAGGTGACTGCCGGTGACAA  1141
             ||||||||||||||||||||||||||||||||||||||||||||||||||||||||||||
Sbjct  1112  AACCCTTGATCTTAGTTGCCAGCATTCAGTTGGGCACTCTAAGGTGACTGCCGGTGACAA  1171

Query  1142  ACCGGAGGAAGGTGGGGATGACGTCAAATCATCATGCCCCTTATGACCTGGGCTACACAC  1201
             ||||||||||||||||||||||||||||||||||||||||||||||||||||||||||||
Sbjct  1172  ACCGGAGGAAGGTGGGGATGACGTCAAATCATCATGCCCCTTATGACCTGGGCTACACAC  1231

Query  1202  GTGCTACAATGGGCAGAACAAAGGGCAGCGAAGCCGCGAGGCTAAGCCAATCCCACAAAT  1261
             ||||||||||||||||||||||||||||||||||||||||||||||||||||||||||||
Sbjct  1232  GTGCTACAATGGGCAGAACAAAGGGCAGCGAAGCCGCGAGGCTAAGCCAATCCCACAAAT  1291

Query  1262  CTGTTCTCAGTTCGGATCGCAGTCTGCAACTCGACTGCGTGAAGCTGGAATCGCTAGTAA  1321
             ||||||||||||||||||||||||||||||||||||||||||||||||||||||||||||
Sbjct  1292  CTGTTCTCAGTTCGGATCGCAGTCTGCAACTCGACTGCGTGAAGCTGGAATCGCTAGTAA  1351

Query  1322  TCGCGGATCAGCATGCCGCGGTGAATACGTTCCCGGGCCTTGTACACACCGCCCGTCACA  1381
             ||||||||||||||||||||||||||||||||||||||||||||||||||||||||||||
Sbjct  1352  TCGCGGATCAGCATGCCGCGGTGAATACGTTCCCGGGCCTTGTACACACCGCCCGTCACA  1411

Query  1382  CCACGAGAGTTTGTAACACCCGAAGTCG  1409
             ||||||||||||||||||||||||||||
```

Sbjct 1412 CCACGAGAGTTTGTAACACCCGAAGTCG 1439

Taxonomy

Reports

- ### Lineage

Organism	Blast Name	Score	Number of Hits	Description
Bacillaceae	firmicutes		103	
.Bacillus	firmicutes		76	
..Bacillus subtilis group	firmicutes		43	
...Bacillus licheniformis	firmicutes	2553	5	**Bacillus licheniformis hits**
...Bacillus sonorensis	firmicutes	2525	2	**Bacillus sonorensis hits**
...Bacillus amyloliquefaciens	firmicutes	2435	4	**Bacillus amyloliquefaciens hits**
...Bacillus atrophaeus	firmicutes	2431	2	**Bacillus atrophaeus hits**
...Bacillus subtilis subsp. subtilis	firmicutes	2423	1	**Bacillus subtilis subsp. subtilis hits**
...Bacillus vallismortis	firmicutes	2420	2	**Bacillus vallismortis hits**
...Bacillus inaquosorum	firmicutes	2418	1	**Bacillus inaquosorum hits**
...Bacillus stercoris	firmicutes	2418	2	**Bacillus stercoris hits**
...Bacillus subtilis	firmicutes	2412	7	**Bacillus subtilis hits**
...Bacillus spizizenii	firmicutes	2412	2	**Bacillus spizizenii hits**
...Bacillus tequilensis	firmicutes	2412	1	**Bacillus tequilensis hits**
...Bacillus velezensis	firmicutes	2410	2	**Bacillus velezensis hits**
...Bacillus halotolerans	firmicutes	2407	6	**Bacillus halotolerans hits**
...Bacillus mojavensis	firmicutes	2407	3	**Bacillus mojavensis hits**
...Bacillus siamensis KCTC 13613	firmicutes	2403	1	**Bacillus siamensis KCTC 13613 hits**
...Bacillus amyloliquefaciens DSM 7 = ATCC 23350	firmicutes	2335	1	**Bacillus amyloliquefaciens DSM 7 = ATCC 23350 hits**
...Bacillus paralicheniformis	firmicutes	2228	1	**Bacillus paralicheniformis hits**
..Bacillus haynesii	firmicutes	2536	1	**Bacillus haynesii hits**
..Bacillus aerius	firmicutes	2508	2	**Bacillus aerius hits**
..Bacillus swezeyi	firmicutes	2473	1	**Bacillus swezeyi hits**
..Bacillus nakamurai	firmicutes	2435	1	**Bacillus nakamurai hits**
..Bacillus cabrialesii	firmicutes	2418	1	**Bacillus cabrialesii hits**
..Bacillus rugosus	firmicutes	2412	2	**Bacillus rugosus hits**
..Bacillus nematocida	firmicutes	2403	1	**Bacillus nematocida hits**
..Bacillus piscis	firmicutes	2344	1	**Bacillus piscis hits**
..Bacillus altitudinis 41KF2b	firmicutes	2290	1	**Bacillus altitudinis 41KF2b hits**
..Bacillus stratosphericus	firmicutes	2290	2	**Bacillus stratosphericus hits**
..Bacillus aerophilus	firmicutes	2290	1	**Bacillus aerophilus hits**
..Bacillus xiamenensis	firmicutes	2285	1	**Bacillus xiamenensis hits**
..Bacillus safensis	firmicutes	2279	1	**Bacillus safensis hits**
..Bacillus capparidis	firmicutes	2278	1	**Bacillus capparidis hits**
..Bacillus pumilus	firmicutes	2274	3	**Bacillus pumilus hits**
..Bacillus safensis FO-36b	firmicutes	2272	1	**Bacillus safensis FO-36b hits**
..Bacillus gobiensis	firmicutes	2270	1	**Bacillus gobiensis hits**
..Bacillus zhangzhouensis	firmicutes	2268	1	**Bacillus zhangzhouensis hits**
..Bacillus australimaris	firmicutes	2268	1	**Bacillus australimaris hits**
..Bacillus haikouensis	firmicutes	2265	1	**Bacillus haikouensis hits**
..Bacillus salacetis	firmicutes	2242	1	**Bacillus salacetis hits**
..Bacillus carboniphilus	firmicutes	2233	1	**Bacillus carboniphilus hits**
..Bacillus timonensis	firmicutes	2207	1	**Bacillus timonensis hits**
..Bacillus isabeliae	firmicutes	2183	1	**Bacillus isabeliae hits**

..Bacillus salis	firmicutes	2178	1	Bacillus salis hits
..Bacillus mesophilum	firmicutes	2176	1	Bacillus mesophilum hits
..Bacillus nitroreducens	firmicutes	2174	1	Bacillus nitroreducens hits
..Bacillus oleivorans	firmicutes	2172	1	Bacillus oleivorans hits
.Calidifontibacillus erzurumensis	firmicutes	2407	2	Calidifontibacillus erzurumensis hits
.Mesobacillus aurantius	firmicutes	2257	1	Mesobacillus aurantius hits
.Rossellomorea aquimaris	firmicutes	2250	1	Rossellomorea aquimaris hits
.Rossellomorea marisflavi	firmicutes	2237	2	Rossellomorea marisflavi hits
.Rossellomorea arthrocnemi	firmicutes	2233	1	Rossellomorea arthrocnemi hits
.Heyndrickxia acidicola	firmicutes	2224	1	Heyndrickxia acidicola hits
.Cytobacillus massiliigabonensis	firmicutes	2215	1	Cytobacillus massiliigabonensis hits
.Litchfieldia sinesaloumensis	firmicutes	2206	1	Litchfieldia sinesaloumensis hits
.Mangrovibacillus cuniculi	firmicutes	2204	1	Mangrovibacillus cuniculi hits
.Falsibacillus albus	firmicutes	2202	1	Falsibacillus albus hits
.Heyndrickxia shackletonii	firmicutes	2202	1	Heyndrickxia shackletonii hits
.Fredinandcohnia onubensis	firmicutes	2191	1	Fredinandcohnia onubensis hits
.Heyndrickxia camelliae	firmicutes	2191	1	Heyndrickxia camelliae hits
.Metabacillus galliciensis	firmicutes	2187	1	Metabacillus galliciensis hits
.Fredinandcohnia salidurans	firmicutes	2183	1	Fredinandcohnia salidurans hits
.Metabacillus herbersteinensis	firmicutes	2180	1	Metabacillus herbersteinensis hits
.Fredinandcohnia humi	firmicutes	2180	1	Fredinandcohnia humi hits
.Cytobacillus firmus	firmicutes	2180	2	Cytobacillus firmus hits
.Metabacillus idriensis	firmicutes	2178	1	Metabacillus idriensis hits
.Mesobacillus foraminis	firmicutes	2176	1	Mesobacillus foraminis hits
.Cytobacillus gottheilii	firmicutes	2174	1	Cytobacillus gottheilii hits
.Cytobacillus praedii	firmicutes	2174	1	Cytobacillus praedii hits
.Heyndrickxia sporothermodurans	firmicutes	2174	1	Heyndrickxia sporothermodurans hits
.Rossellomorea vietnamensis	firmicutes	2170	1	Rossellomorea vietnamensis hits

- **Organism**

Description	Score	E value	Accession
Bacillus licheniformis [firmicutes]			
**Bacillus licheniformis strain BCRC 11702 16S ribosomal RNA, partial sequence**	2553	0.0	NR_116023
**Bacillus licheniformis strain DSM 13 16S ribosomal RNA, partial sequence**	2553	0.0	NR_118996
**Bacillus licheniformis strain NBRC 12200 16S ribosomal RNA, partial sequence**	2547	0.0	NR_113588
**Bacillus licheniformis strain ATCC 14580 16S ribosomal RNA, partial sequence**	2542	0.0	NR_074923
**Bacillus licheniformis strain NCDO 1772 16S ribosomal RNA, partial sequence**	2375	0.0	NR_118959
Bacillus haynesii [firmicutes]			
**Bacillus haynesii strain NRRL B-41327 16S ribosomal RNA, partial sequence**	2536	0.0	NR_157609
Bacillus sonorensis [firmicutes]			
**Bacillus sonorensis strain NBRC 101234 16S ribosomal RNA, partial sequence**	2525	0.0	NR_113993
**Bacillus sonorensis strain NRRL B-23154 16S ribosomal RNA, partial sequence**	2507	0.0	NR_025130
Bacillus aerius [firmicutes]			
**Bacillus aerius strain 24K 16S ribosomal RNA, partial sequence**	2508	0.0	NR_042338
**Bacillus aerius strain 24K 16S ribosomal RNA, partial sequence**	2287	0.0	NR_118439
Bacillus swezeyi [firmicutes]			
**Bacillus swezeyi strain NRRL B-41294 16S ribosomal RNA, partial sequence**	2473	0.0	NR_157608
Bacillus amyloliquefaciens [firmicutes]			
**Bacillus amyloliquefaciens strain BCRC 11601 16S ribosomal RNA, partial sequence**	2435	0.0	NR_116022
**Bacillus amyloliquefaciens strain NBRC 15535 16S ribosomal RNA, partial sequence**	2431	0.0	NR_112685
**Bacillus amyloliquefaciens strain NBRC 15535 16S ribosomal RNA, partial sequence**	2429	0.0	NR_041455

Description	Score	E value	Accession
Bacillus amyloliquefaciens strain MPA 1034 16S ribosomal RNA, partial sequence	2429	0.0	NR_117946
Bacillus nakamurai [firmicutes]			
Bacillus nakamurai strain NRRL B-41091 16S ribosomal RNA, partial sequence	2435	0.0	NR_151897
Bacillus atrophaeus [firmicutes]			
Bacillus atrophaeus strain NBRC 15539 16S ribosomal RNA, partial sequence	2431	0.0	NR_112723
Bacillus atrophaeus strain JCM 9070 16S ribosomal RNA, partial sequence	2429	0.0	NR_024689
Bacillus subtilis subsp. subtilis [firmicutes]			
Bacillus subtilis subsp. subtilis strain 168 16S ribosomal RNA, complete sequence	2423	0.0	NR_102783
Bacillus vallismortis [firmicutes]			
Bacillus vallismortis strain NBRC 101236 16S ribosomal RNA, partial sequence	2420	0.0	NR_113994
Bacillus vallismortis strain DSM 11031 16S ribosomal RNA, partial sequence	2418	0.0	NR_024696
Bacillus inaquosorum [firmicutes]			
Bacillus inaquosorum strain BGSC 3A28 16S ribosomal RNA, partial sequence	2418	0.0	NR_104873
Bacillus stercoris [firmicutes]			
Bacillus stercoris strain D7XPN1 16S ribosomal RNA, partial sequence	2418	0.0	NR_181952
Bacillus stercoris strain JCM 30051 16S ribosomal RNA, partial sequence	2418	0.0	NR_180796
Bacillus cabrialesii [firmicutes]			
Bacillus cabrialesii strain TE3 16S ribosomal RNA, complete sequence	2418	0.0	NR_180419
Bacillus subtilis [firmicutes]			
Bacillus subtilis strain JCM 1465 16S ribosomal RNA, partial sequence	2412	0.0	NR_113265
Bacillus subtilis strain NBRC 13719 16S ribosomal RNA, partial sequence	2412	0.0	NR_112629
Bacillus subtilis strain DSM 10 16S ribosomal RNA, partial sequence	2412	0.0	NR_027552
Bacillus subtilis strain BCRC 10255 16S ribosomal RNA, partial sequence	2407	0.0	NR_116017
Bacillus subtilis strain IAM 12118 16S ribosomal RNA, complete sequence	2407	0.0	NR_112116
Bacillus subtilis strain NCDO 1769 16S ribosomal RNA, partial sequence	2351	0.0	NR_118972
Bacillus subtilis strain SBMP4 16S ribosomal RNA, partial sequence	2331	0.0	NR_118383
Bacillus spizizenii [firmicutes]			
Bacillus spizizenii strain NBRC 101239 16S ribosomal RNA, partial sequence	2412	0.0	NR_112686
Bacillus spizizenii strain NRRL B-23049 16S ribosomal RNA, partial sequence	2399	0.0	NR_024931
Bacillus rugosus [firmicutes]			
Bacillus rugosus strain SPB7 16S ribosomal RNA, partial sequence	2412	0.0	NR_181236
Bacillus rugosus strain SPB7 16S ribosomal RNA, partial sequence	2392	0.0	NR_180415
Bacillus tequilensis [firmicutes]			
Bacillus tequilensis strain 10b 16S ribosomal RNA, partial sequence	2412	0.0	NR_104919
Bacillus velezensis [firmicutes]			
Bacillus velezensis strain CBMB205 16S ribosomal RNA, partial sequence	2410	0.0	NR_116240
Bacillus velezensis strain FZB42 16S ribosomal RNA, complete sequence	2407	0.0	NR_075005
Bacillus halotolerans [firmicutes]			
Bacillus halotolerans strain LMG 22476 16S ribosomal RNA, partial sequence	2407	0.0	NR_115929
Bacillus halotolerans strain CECT 5687 16S ribosomal RNA, partial sequence	2407	0.0	NR_115930
Bacillus halotolerans strain LMG 22477 16S ribosomal RNA, partial sequence	2407	0.0	NR_115931
Bacillus halotolerans strain DSM 8802 16S ribosomal RNA, partial sequence	2407	0.0	NR_115063
Bacillus halotolerans strain CR-95 16S ribosomal RNA, partial sequence	2403	0.0	NR_115282
Bacillus halotolerans strain CR-119 16S ribosomal RNA, partial sequence	2359	0.0	NR_115283
Bacillus mojavensis [firmicutes]			
Bacillus mojavensis strain NBRC 15718 16S ribosomal RNA, partial sequence	2407	0.0	NR_112725
Bacillus mojavensis strain IFO 15718 16S ribosomal RNA, partial sequence	2407	0.0	NR_024693
Bacillus mojavensis strain IFO 15718 16S ribosomal RNA, partial sequence	2398	0.0	NR_118290
Calidifontibacillus erzurumensis [firmicutes]			
Calidifontibacillus erzurumensis strain P2 16S ribosomal RNA, partial sequence	2407	0.0	NR_180225

Description	Score	E value	Accession
Calidifontibacillus erzurumensis strain P2 16S ribosomal RNA, partial sequence	2401	0.0	NR_178988
Bacillus nematocida [firmicutes]			
Bacillus nematocida strain B-16 16S ribosomal RNA, partial sequence	2403	0.0	NR_115325
Bacillus siamensis KCTC 13613 [firmicutes]			
Bacillus siamensis KCTC 13613 strain PD-A10 16S ribosomal RNA, partial sequence	2403	0.0	NR_117274
Bacillus piscis [firmicutes]			
Bacillus piscis strain 16MFT21 16S ribosomal RNA, partial sequence	2344	0.0	NR_165685
Bacillus amyloliquefaciens DSM 7 = ATCC 23350 [firmicutes]			
Bacillus amyloliquefaciens DSM 7 = ATCC 23350 16S ribosomal RNA, partial sequence	2335	0.0	NR_118950
Bacillus altitudinis 41KF2b [firmicutes]			
Bacillus altitudinis 41KF2b 16S ribosomal RNA, partial sequence	2290	0.0	NR_042337
Bacillus stratosphericus [firmicutes]			
Bacillus stratosphericus strain 41KF2a 16S ribosomal RNA, partial sequence	2290	0.0	NR_042336
Bacillus stratosphericus strain 41KF2a 16S ribosomal RNA, partial sequence	2281	0.0	NR_118441
Bacillus aerophilus [firmicutes]			
Bacillus aerophilus strain 28K 16S ribosomal RNA, partial sequence	2290	0.0	NR_042339
Bacillus xiamenensis [firmicutes]			
Bacillus xiamenensis strain MCCC 1A00008 16S ribosomal RNA, partial sequence	2285	0.0	NR_148244
Bacillus safensis [firmicutes]			
Bacillus safensis strain NBRC 100820 16S ribosomal RNA, partial sequence	2279	0.0	NR_113945
Bacillus capparidis [firmicutes]			
Bacillus capparidis strain EGI 6500252 16S ribosomal RNA, partial sequence	2278	0.0	NR_156073
Bacillus pumilus [firmicutes]			
Bacillus pumilus strain NBRC 12092 16S ribosomal RNA, partial sequence	2274	0.0	NR_112637
Bacillus pumilus strain ATCC 7061 16S ribosomal RNA, partial sequence	2266	0.0	NR_043242
Bacillus pumilus strain SBMP2 16S ribosomal RNA, partial sequence	2176	0.0	NR_118381
Bacillus safensis FO-36b [firmicutes]			
Bacillus safensis FO-36b 16S ribosomal RNA, partial sequence	2272	0.0	NR_041794
Bacillus gobiensis [firmicutes]			
Bacillus gobiensis strain FJAT-4402 16S ribosomal RNA, partial sequence	2270	0.0	NR_147766
Bacillus zhangzhouensis [firmicutes]			
Bacillus zhangzhouensis strain MCCC 1A08372 16S ribosomal RNA, partial sequence	2268	0.0	NR_148786
Bacillus australimaris [firmicutes]			
Bacillus australimaris strain MCCC 1A05787 16S ribosomal RNA, partial sequence	2268	0.0	NR_148787
Bacillus haikouensis [firmicutes]			
Bacillus haikouensis strain C-89 16S ribosomal RNA, partial sequence	2265	0.0	NR_148273
Mesobacillus aurantius [firmicutes]			
Mesobacillus aurantius strain JC1013 16S ribosomal RNA, partial sequence	2257	0.0	NR_180197
Rossellomorea aquimaris [firmicutes]			
Rossellomorea aquimaris strain TF-12 16S ribosomal RNA, partial sequence	2250	0.0	NR_025241
Bacillus salacetis [firmicutes]			
Bacillus salacetis strain SKP7-4 16S ribosomal RNA, partial sequence	2242	0.0	NR_179253
Rossellomorea marisflavi [firmicutes]			
Rossellomorea marisflavi strain TF-11 16S ribosomal RNA, partial sequence	2237	0.0	NR_025240
Rossellomorea marisflavi strain TF-11 16S ribosomal RNA, partial sequence	2235	0.0	NR_118437
Rossellomorea arthrocnemi [firmicutes]			
Rossellomorea arthrocnemi strain EAR8 16S ribosomal RNA, partial sequence	2233	0.0	NR_181775
Bacillus carboniphilus [firmicutes]			
Bacillus carboniphilus strain JCM9731 16S ribosomal RNA, partial sequence	2233	0.0	NR_024690
Bacillus paralicheniformis [firmicutes]			

Description	Score	E value	Accession
Bacillus paralicheniformis strain KJ-16 16S ribosomal RNA, partial sequence	2228	0.0	NR_137421
Heyndrickxia acidicola [firmicutes]			
Heyndrickxia acidicola strain 105-2 16S ribosomal RNA, partial sequence	2224	0.0	NR_041942
Cytobacillus massiliigabonensis [firmicutes]			
Cytobacillus massiliigabonensis strain Marseille-P2639 16S ribosomal RNA, partial sequence	2215	0.0	NR_179554
Bacillus timonensis [firmicutes]			
Bacillus timonensis strain 10403023 16S ribosomal RNA, partial sequence	2207	0.0	NR_133024
Bacillus sinesaloumensis [firmicutes]			
Litchfieldia sinesaloumensis strain Marseille-P3516 16S ribosomal RNA, partial sequence	2206	0.0	NR_147383
Mangrovibacillus cuniculi [firmicutes]			
Mangrovibacillus cuniculi strain R1DC41 16S ribosomal RNA, partial sequence	2204	0.0	NR_181118
Falsibacillus albus [firmicutes]			
Falsibacillus albus strain GY 10110 16S ribosomal RNA, partial sequence	2202	0.0	NR_171509
Heyndrickxia shackletonii [firmicutes]			
Heyndrickxia shackletonii strain LMG 18435 16S ribosomal RNA, partial sequence	2202	0.0	NR_025373
Fredinandcohnia onubensis [firmicutes]			
Fredinandcohnia onubensis strain 0911MAR22V3 16S ribosomal RNA, partial sequence	2191	0.0	NR_149252
Heyndrickxia camelliae [firmicutes]			
Heyndrickxia camelliae strain 7578-1 16S ribosomal RNA, partial sequence	2191	0.0	NR_159341
Metabacillus galliciensis [firmicutes]			
Metabacillus galliciensis strain BFLP-1 16S ribosomal RNA, partial sequence	2187	0.0	NR_116886
Bacillus isabeliae [firmicutes]			
Bacillus isabeliae strain CVS-8 16S ribosomal RNA, partial sequence	2183	0.0	NR_042619
Fredinandcohnia salidurans [firmicutes]			
Fredinandcohnia salidurans strain KNUC7312 16S ribosomal RNA, partial sequence	2183	0.0	NR_179035
Metabacillus herbersteinensis [firmicutes]			
Metabacillus herbersteinensis strain D-1,5 16S ribosomal RNA, partial sequence	2180	0.0	NR_042286
Fredinandcohnia humi [firmicutes]			
Fredinandcohnia humi strain LMG 22167 16S ribosomal RNA, partial sequence	2180	0.0	NR_025626
Cytobacillus firmus [firmicutes]			
Cytobacillus firmus strain IAM 12464 16S ribosomal RNA, partial sequence	2180	0.0	NR_025842
Cytobacillus firmus strain NBRC 15306 16S ribosomal RNA, partial sequence	2174	0.0	NR_112635
Bacillus salis [firmicutes]			
Bacillus salis strain ES3 16S ribosomal RNA, partial sequence	2178	0.0	NR_179406
Metabacillus idriensis [firmicutes]			
Metabacillus idriensis strain SMC 4352-2 16S ribosomal RNA, partial sequence	2178	0.0	NR_043268
Bacillus mesophilum [firmicutes]			
Bacillus mesophilum strain IITR-54 16S ribosomal RNA, partial sequence	2176	0.0	NR_178489
Mesobacillus foraminis [firmicutes]			
Mesobacillus foraminis strain CV53 16S ribosomal RNA, partial sequence	2176	0.0	NR_042274
Cytobacillus gottheilii [firmicutes]			
Cytobacillus gottheilii strain WCC 4585 16S ribosomal RNA, partial sequence	2174	0.0	NR_108491
Cytobacillus praedii [firmicutes]			
Cytobacillus praedii strain FJAT-25547 16S ribosomal RNA, partial sequence	2174	0.0	NR_157745
Bacillus nitroreducens [firmicutes]			
Bacillus nitroreducens strain GSS08 16S ribosomal RNA, partial sequence	2174	0.0	NR_178876
Heyndrickxia sporothermodurans [firmicutes]			
Heyndrickxia sporothermodurans strain M215 16S ribosomal RNA, partial sequence	2174	0.0	NR_026010
Bacillus oleivorans [firmicutes]			
Bacillus oleivorans strain JC228 16S ribosomal RNA, partial sequence	2172	0.0	NR_134703

Description	Score	E value	Accession
Rossellomorea vietnamensis [firmicutes]			
**Rossellomorea vietnamensis strain 15-1 16S ribosomal RNA, partial sequence**	2170	0.0	**NR_024808**

- ## Taxonomy

Taxonomy	Number of hits	Number of Organisms	Description
Bacillaceae	103	69	
. Bacillus	76	45	
.. Bacillus subtilis group	43	17	
... Bacillus licheniformis	5	1	**Bacillus licheniformis hits**
... Bacillus sonorensis	2	1	**Bacillus sonorensis hits**
... Bacillus amyloliquefaciens group	8	4	
.... Bacillus amyloliquefaciens	4	2	**Bacillus amyloliquefaciens hits**
..... Bacillus amyloliquefaciens DSM 7 = ATCC 23350	1	1	**Bacillus amyloliquefaciens DSM 7 = ATCC 23350 hits**
.... Bacillus velezensis	2	1	**Bacillus velezensis hits**
.... Bacillus siamensis KCTC 13613	1	1	**Bacillus siamensis KCTC 13613 hits**
... Bacillus atrophaeus	2	1	**Bacillus atrophaeus hits**
... Bacillus subtilis	7	2	**Bacillus subtilis hits**
.... Bacillus subtilis subsp. subtilis	1	1	**Bacillus subtilis subsp. subtilis hits**
... Bacillus vallismortis	2	1	**Bacillus vallismortis hits**
... Bacillus inaquosorum	1	1	**Bacillus inaquosorum hits**
... Bacillus stercoris	2	1	**Bacillus stercoris hits**
... Bacillus spizizenii	2	1	**Bacillus spizizenii hits**
... Bacillus tequilensis	1	1	**Bacillus tequilensis hits**
... Bacillus mojavensis subgroup	9	2	
.... Bacillus halotolerans	6	1	**Bacillus halotolerans hits**
.... Bacillus mojavensis	3	1	**Bacillus mojavensis hits**
... Bacillus paralicheniformis	1	1	**Bacillus paralicheniformis hits**
.. Bacillus haynesii	1	1	**Bacillus haynesii hits**
.. Bacillus aerius	2	1	**Bacillus aerius hits**
.. Bacillus swezeyi	1	1	**Bacillus swezeyi hits**
.. Bacillus nakamurai	1	1	**Bacillus nakamurai hits**
.. Bacillus cabrialesii	1	1	**Bacillus cabrialesii hits**
.. Bacillus rugosus	2	1	**Bacillus rugosus hits**
.. Bacillus nematocida	1	1	**Bacillus nematocida hits**
.. Bacillus piscis	1	1	**Bacillus piscis hits**
.. Bacillus altitudinis complex	4	3	
... Bacillus altitudinis 41KF2b	1	1	**Bacillus altitudinis 41KF2b hits**
... Bacillus stratosphericus	2	1	**Bacillus stratosphericus hits**
... Bacillus aerophilus	1	1	**Bacillus aerophilus hits**
.. Bacillus xiamenensis	1	1	**Bacillus xiamenensis hits**
.. Bacillus safensis	1	2	**Bacillus safensis hits**
... Bacillus safensis FO-36b	1	1	**Bacillus safensis FO-36b hits**
.. Bacillus capparidis	1	1	**Bacillus capparidis hits**
.. Bacillus pumilus	3	1	**Bacillus pumilus hits**
.. Bacillus gobiensis	1	1	**Bacillus gobiensis hits**
.. Bacillus zhangzhouensis	1	1	**Bacillus zhangzhouensis hits**
.. Bacillus australimaris	1	1	**Bacillus australimaris hits**
.. Bacillus haikouensis	1	1	**Bacillus haikouensis hits**
.. Bacillus salacetis	1	1	**Bacillus salacetis hits**

.. Bacillus carboniphilus	1	1	Bacillus carboniphilus hits
.. Bacillus timonensis	1	1	Bacillus timonensis hits
.. Bacillus isabeliae	1	1	Bacillus isabeliae hits
.. Bacillus salis	1	1	Bacillus salis hits
.. Bacillus mesophilum	1	1	Bacillus mesophilum hits
.. Bacillus nitroreducens	1	1	Bacillus nitroreducens hits
.. Bacillus oleivorans	1	1	Bacillus oleivorans hits
. Calidifontibacillus erzurumensis	2	1	Calidifontibacillus erzurumensis hits
. Mesobacillus	2	2	
.. Mesobacillus aurantius	1	1	Mesobacillus aurantius hits
.. Mesobacillus foraminis	1	1	Mesobacillus foraminis hits
. Rossellomorea	5	4	
.. Rossellomorea aquimaris	1	1	Rossellomorea aquimaris hits
.. Rossellomorea marisflavi	2	1	Rossellomorea marisflavi hits
.. Rossellomorea arthrocnemi	1	1	Rossellomorea arthrocnemi hits
.. Rossellomorea vietnamensis	1	1	Rossellomorea vietnamensis hits
. Heyndrickxia	4	4	
.. Heyndrickxia acidicola	1	1	Heyndrickxia acidicola hits
.. Heyndrickxia shackletonii	1	1	Heyndrickxia shackletonii hits
.. Heyndrickxia camelliae	1	1	Heyndrickxia camelliae hits
.. Heyndrickxia sporothermodurans	1	1	Heyndrickxia sporothermodurans hits
. Cytobacillus	5	4	
.. Cytobacillus massiliigabonensis	1	1	Cytobacillus massiliigabonensis hits
.. Cytobacillus firmus	2	1	Cytobacillus firmus hits
.. Cytobacillus gottheilii	1	1	Cytobacillus gottheilii hits
.. Cytobacillus praedii	1	1	Cytobacillus praedii hits
. Litchfieldia sinesaloumensis	1	1	Litchfieldia sinesaloumensis hits
. Mangrovibacillus cuniculi	1	1	Mangrovibacillus cuniculi hits
. Falsibacillus albus	1	1	Falsibacillus albus hits
. Fredinandcohnia	3	3	
.. Fredinandcohnia onubensis	1	1	Fredinandcohnia onubensis hits
.. Fredinandcohnia salidurans	1	1	Fredinandcohnia salidurans hits
.. Fredinandcohnia humi	1	1	Fredinandcohnia humi hits
. Metabacillus	3	3	
.. Metabacillus galliciensis	1	1	Metabacillus galliciensis hits
.. Metabacillus herbersteinensis	1	1	Metabacillus herbersteinensis hits
.. Metabacillus idriensis	1	1	Metabacillus idriensis hits
